# Macrophage polarization impacts tunneling nanotube formation and intercellular organelle trafficking

**DOI:** 10.1038/s41598-019-50971-x

**Published:** 2019-10-10

**Authors:** Spencer Goodman, Swati Naphade, Meisha Khan, Jay Sharma, Stephanie Cherqui

**Affiliations:** Department of Pediatrics, Division of Genetics, University of California, San Diego, La Jolla, California USA

**Keywords:** Cellular imaging, Membrane trafficking, Lysosomes, Gene therapy, Monocytes and macrophages

## Abstract

Tunneling nanotubes (TNTs) are cellular extensions enabling cytosol-to-cytosol intercellular interaction between numerous cell types including macrophages. Previous studies of hematopoietic stem and progenitor cell (HSPC) transplantation for the lysosomal storage disorder cystinosis have shown that HSPC-derived macrophages form TNTs to deliver cystinosin-bearing lysosomes to cystinotic cells, leading to tissue preservation. Here, we explored if macrophage polarization to either proinflammatory M1-like M(LPS/IFNγ) or anti-inflammatory M2-like M(IL-4/IL-10) affected TNT-like protrusion formation, intercellular transport and, ultimately, the efficacy of cystinosis prevention. We designed new automated image processing algorithms used to demonstrate that LPS/IFNγ polarization decreased bone marrow-derived macrophages (BMDMs) formation of protrusions, some of which displayed characteristics of TNTs, including cytoskeletal structure, 3D morphology and size. In contrast, co-culture of macrophages with cystinotic fibroblasts yielded more frequent and larger protrusions, as well as increased lysosomal and mitochondrial intercellular trafficking to the diseased fibroblasts. Unexpectedly, we observed normal protrusion formation and therapeutic efficacy following disruption of anti-inflammatory IL-4/IL-10 polarization *in vivo* by transplantation of HSPCs isolated from the *Rac2*^−/−^ mouse model. Altogether, we developed unbiased image quantification systems that probe mechanistic aspects of TNT formation and function *in vitro*, while HSPC transplantation into cystinotic mice provides a complex *in vivo* disease model. While the differences between polarization cell culture and mouse models exemplify the oversimplicity of *in vitro* cytokine treatment, they simultaneously demonstrate the utility of our co-culture model which recapitulates the *in vivo* phenomenon of diseased cystinotic cells stimulating thicker TNT formation and intercellular trafficking from macrophages. Ultimately, we can use both approaches to expand the utility of TNT-like protrusions as a delivery system for regenerative medicine.

## Introduction

Intercellular communication is essential to maintain and potentially restore homeostasis in multicellular organisms. Tunneling nanotubes (TNTs) are one interaction pathway consisting of long actin-rich membranous extensions capable of forming cytosolic connections between distant cells^1–3^. TNTs have been observed *in vitro* and *in vivo* facilitating transfer of molecular cargos ranging from electrical signals to organelles to pathogens^4–6^. Mechanistic investigations of hematopoietic stem and progenitor cell (HSPC) transplantation therapy for the lysosomal storage disorder cystinosis revealed HSPC-derived macrophages deliver functional lysosomal protein cystinosin through TNTs to diseased tissue, resulting in lifelong prevention of disease progression in the mouse model^7–9^.

Cystinosin is ubiquitously expressed within the lysosomal membrane to export cystine into the cytosol^10^. However, loss-of-function mutations in the *CTNS* gene cause lysosomal cystine accumulation and crystallization leading to multi-organ failure^11,12^. Current therapies merely ameliorate symptoms and delay disease progression^13^. Our group has pioneered the use of HSPC transplantation as a promising new therapy for cystinosis with translation of this approach into an autologous gene-corrected HSPC transplantation clinical trial for human cystinosis patients underway^14–16^.

Due to cystinosin being a non-secreted transmembrane protein, the ubiquitous prevention of pathogenic cell damage using HSPCs was unexpected until the mechanism of trafficking was shown to involve TNT-mediated delivery of healthy lysosomes from HSPC-derived macrophages to diseased tissue^7^. While many studies have demonstrated how pathogens like HIV^17^ or malignancies like gliomas^18^ can hijack TNTs, the structures also present an intriguing potential delivery system of therapeutic proteins to treat genetic diseases^19^. Following HSPC transplantation, kidney macrophages formed TNTs that crossed the basement membrane and rescued proximal tubule cells by delivering cystinosin-bearing lysosomes^7^. The same mechanism has also been observed in the cornea and thyroid of transplanted cystinotic mice^8,9^, as well as in the X-linked tubulopathy Dent disease^20^. In addition, studies of HSPC transplantation as treatment for the mitochondrial neurodegenerative disorder Friedreich’s Ataxia reveal microglial correction of neurons, potentially through TNTs^21^. Taken together, these data indicate a prolific ability of phagocytic cells to widely disseminate therapeutic molecules via TNT trafficking.

The plasticity of macrophages allows them to fulfill numerous biological functions ranging from proinflammatory roles in both arms of the immune system to immunomodulatory activities vis-à-vis tissue repair, homeostasis and resolution of inflammation. Traditionally, these distinct phenotypic behaviors have been classified as either proinflammatory mediators of a type I immune response (M1 or classical activation) versus. immunomodulatory tissue-remodelers (M2 or alternative)^22,23^. However, this model risks oversimplification of a seamless phenotypic spectrum into a false dichotomy masking *in vivo* macrophage complexity, where these subtypes are dynamically changing in response to the cues received from the microenvironment, and thus never cleanly delineated^24,25^.

In the current study, we investigated macrophage-mediated TNT formation and intercellular trafficking. Using novel image analysis platforms, we report that *in vitro* proinflammatory macrophage stimulation suppressed both TNT-like protrusion formation as well as intercellular organelle trafficking. In contrast, macrophages co-cultured with diseased *Ctns*^−/−^ fibroblasts exhibited increased lysosomal and mitochondrial intercellular trafficking along with more frequent formation of larger protrusions. These data suggest that diseased cells stimulate TNT formation towards a thicker phenotype, which has been shown to more effectively transport organelles such as macrophages and intracellular vesicles^26^. Unexpectedly, *in vivo* enrichment of proinflammatory macrophages showed similar disease rescue following *Rac2*^−/−^ HSPC transplantation in *Ctns*^−/−^ mice compared to wild-type (WT) mice. This discrepancy between mice and co-cultures highlights the well-known dangers of relying solely on *in vitro* polarization models where cytokine stimulation pushes macrophages to non-physiological polarization phenotypes. That said, increased macrophage-derived thicker TNT formation and trafficking activity seen in unstimulated cystinotic co-cultures effectively models *in vivo* observations of enhanced macrophage recruitment and intercellular lysosomal delivery after HSPC transplantation^7^. Using both cells and transplanted mice, we can therefore probe aspects of TNTs induced when stress on a separate cellular population is stimulating macrophage TNT formation in a paracrine fashion. Ultimately, a detailed understanding of these processes could ultimately better explain and enhance the effective delivery of non-secreted genetic products.

## Results

### Macrophage membrane protrusions are suppressed by proinflammatory stimulation but enhanced by co-culture with diseased cells

**Figure 1:**
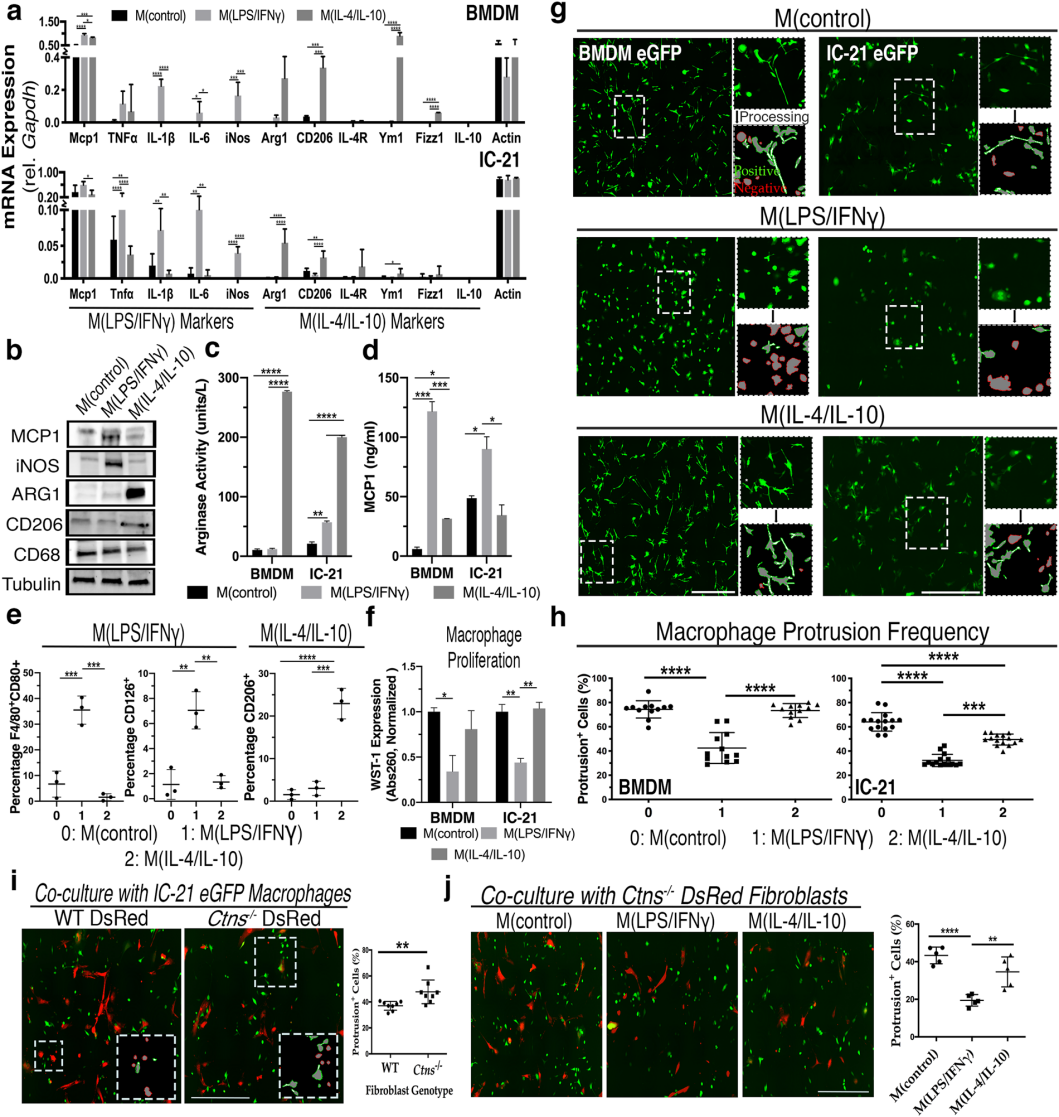
LPS/IFNγ polarization suppresses protrusion formation in BMDMs and IC-21 macrophages. (**a**) Bar graph representing mRNA expression of M(LPS/IFNγ) and M(IL4/IL10) markers relative to housekeeping control *Gapdh* in BMDMs and IC-21 macrophages after 48-hour pro- and anti-inflammatory macrophage polarization compared to non-treated cells (n = 5). (**b**) Representative immunoblots depicting polarization-associated signature protein expression in pro- and anti-inflammatory IC-21 macrophages and untreated controls. MCP1 and iNOS are M(LPS/IFNγ) markers, while ARG1 and CD206 are M(IL-4/IL-10) markers. CD68 is a pan-macrophage marker, and tubulin serves as the loading control (n = 2–4). (**c**) Graph depicts normalized colorimetric measurements of the M(IL-4/IL-10) marker ARG1 enzymatic activity from cell lysate following independent polarization treatments (n = 3). (**d**) Bar graph depicting normalized MCP1 cytokine secretion into media following polarization as measured by ELISA (n = 3). (**e**) Bar graphs representing flow cytometry analysis showing frequency of expression of M(LPS/IFNγ) markers CD80 and CD126 or M(IL-4/IL-10) marker CD206 following BMDM polarization (n = 3). (**f**) Bar graphs presenting normalized colorimetric absorbance values measuring cell proliferation using WST-1 cell proliferation reagent in BMDMs and IC-21 macrophages acquired three days post polarization treatment (n = 3). (**g**) Representative wide-field fluorescent images of control vs. M(LPS/IFNγ)- or M(IL4/IL10)-polarized BMDMs and IC-21 macrophages. Larger micrographs are stitched images of 5 × 5 individual tile. Zoomed inserts demonstrate automated image processing selecting for protrusion-positive (green) or negative (red) cells. See Supplementary Fig. S1 for method description and examples. (**h**) Percentile quantification of protrusion-positive cells in polarized BMDMs and IC-21 macrophages. Each data point in the box-and-whisker histogram is one stitched region with five regions analyzed per experiment (n = 3). (**i**) Representative fluorescent images of eGFP^+^ IC-21 co-cultures grown for three days with either WT (left) or *Ctns*^−/−^ (right) DsRed^+^ fibroblasts. Boxed insert shows automated protrusion detection analysis with percentage of eGFP^+^ protrusion-positive cells shown in histogram (n = 3). (**j**) Representative fluorescent images of polarized eGFP^+^ IC-21 macrophage and *Ctns*^−/−^ DsRed^+^ fibroblast co-cultures. Histogram illustrates percentage of control or M(LPS/IFNγ) and M(IL4/IL10) macrophages with protrusions (n = 3). All scale bars: 500 µM. All bar graphs shown as mean ± standard deviation (SD). *P* values were determined by either one-way ANOVA or student’s *t*-tests. *P < 0.05, **P < 0.01, ***P < 0.001, ****P < 0.0001.

Previous work has uncovered that proinflammatory bone marrow derived macrophages (BMDMs) display a more rounded ameboid morphology compared to unstimulated or anti-inflammatory cells^27^. To investigate tunneling nanotube (TNT) formation in this context, we treated primary murine BMDMs and the immortalized IC-21 peritoneal macrophage cell line with either LPS/IFNγ, directing differentiation towards proinflammatory M1-like cells, M(LPS/IFNγ), or with IL-4/IL-10 for differentiation towards immunomodulatory M2-like macrophages, M(IL-4/IL-10). Using a variety of assays to characterize expression of well-established macrophage polarization markers, we rigorously validated our *in vitro* polarization experimental system. First, we analyzed differentially expressed mRNAs and proteins, observing increased expression of numerous proinflammatory markers such as inducible nitric oxide synthase (*iNos*), interleukin-6 (*Il-6*) and interleukin-1β (*Il-1β*) following LPS/IFNγ treatment, as well as upregulation of anti-inflammatory markers such as *Cd206*, *Ym1* and *Fizz1* after IL-4/IL-10 stimulation (Fig. 1a,b, Supplementary Fig. S1)^28,29^. Furthermore, M(IL-4/IL-10) displayed greater enzymatic activity of arginase-1 (ARG1), an important biosynthetic regulator of tissue repair, while M(LPS/IFNγ) increased extracellular secretion of monocyte chemoattractant protein 1 (MCP1) (Fig. 1c,d), a potent proinflammatory chemokine that regulates migration and infiltration of macrophages^30^. Finally, polarized BMDMs were analyzed by flow cytometry where we observed expected changes in expression of proinflammatory markers CD80 and CD126 or anti-inflammatory CD206 (Fig. 1e)^31^. To assess macrophage viability following polarization, we also measured cell proliferation using WST-1, which decreased following LPS/IFNγ but not IL-4/IL-10 stimulation in both BMDMs (*P* = 0.0015) and IC-21 macrophages (*P* < 0.0001) compared to non-treated controls (Fig. 1f). In contrast, apoptosis measured by Annexin V and Propidium Iodide-stained cells did not vary between treatment groups (data not shown). Taken together, *in vitro* treatment yields the anticipated changes in marker expression at both the mRNA and protein levels, confirming that our system replicates the expected polarization findings.

Upon imaging of treated cells, we also observed the expected shift of M(LPS/IFNγ) morphology to a more rounded phenotype in both BMDMs and IC-21 (Fig. 1g). We developed an image analysis workflow using ImagePro Premier to automatically detect and quantify fluorescent membrane protrusions using customizable morphological filtration based on characteristics such as length, width and circularity (Supplementary Fig. S2). By analyzing larger stitched images acquired using the wide-field Keyence fluorescence microscope, our high-throughput method enables automated, rapid and unbiased analysis of fluorescent cellular protrusions from dozens to hundreds of cells at once. Using this algorithm, we observed a significant reduction of approximately 50% in the protrusion frequency of BMDMs and IC-21 macrophages following LPS/IFNγ stimulation compared to non-treated macrophages (*P* < 0.001) (Fig. 1h). In contrast, M(IL-4/IL-10) BMDM protrusion frequency was statistically indistinguishable from control, while IC-21 displayed a modest decrease.

We sought to extend on our protrusion frequency findings by stimulating BMDMs with each cytokine or stimulant individually. We observe that either LPS or IFNγ by themselves are equally effective as when combined to reduce protrusion formation compared to untreated or M(IL-4/IL-10) (Supplementary Fig. S3). Furthermore, just as M(IL-4/IL-10) BMDMs, we again detected no difference in protrusion frequency for either M(IL-4) or M(IL-10) BMDM relative to untreated controls. Recent evidence suggests that tuberculosis infection triggers an increase in M(IL-10) macrophage TNT formation, but direct stimulation of BMDMs with IL-10 does not seem to recapitulate this phenotype^32^.

We previously reported that co-culture of IC-21 macrophages with murine cystinotic fibroblasts appeared to increase the formation of TNTs^7^. We replicated these experiments using our new automated imaging system and observed an increase in membrane protrusion formation in IC-21 macrophages co-cultured with diseased murine *Ctns*^−/−^ fibroblasts compared to wildtype fibroblasts (*P* = 0.007) (Fig. 1i). Proinflammatory stimulation of these co-cultures yielded a reduction in macrophage-derived protrusion formation with no obvious morphological changes in fibroblasts (Fig. 1j), confirming our previous data (Fig. 1h). Taken together, these data indicate proinflammatory M(LPS/IFNγ) macrophages generate fewer membrane protrusions than the immunomodulatory M(IL-4/IL-10) cells, and that diseased cystinotic cells enhanced protrusion formation in macrophages upon co-culture.

### Macrophage-derived membrane protrusions resemble tunneling nanotubes

**Figure 2:**
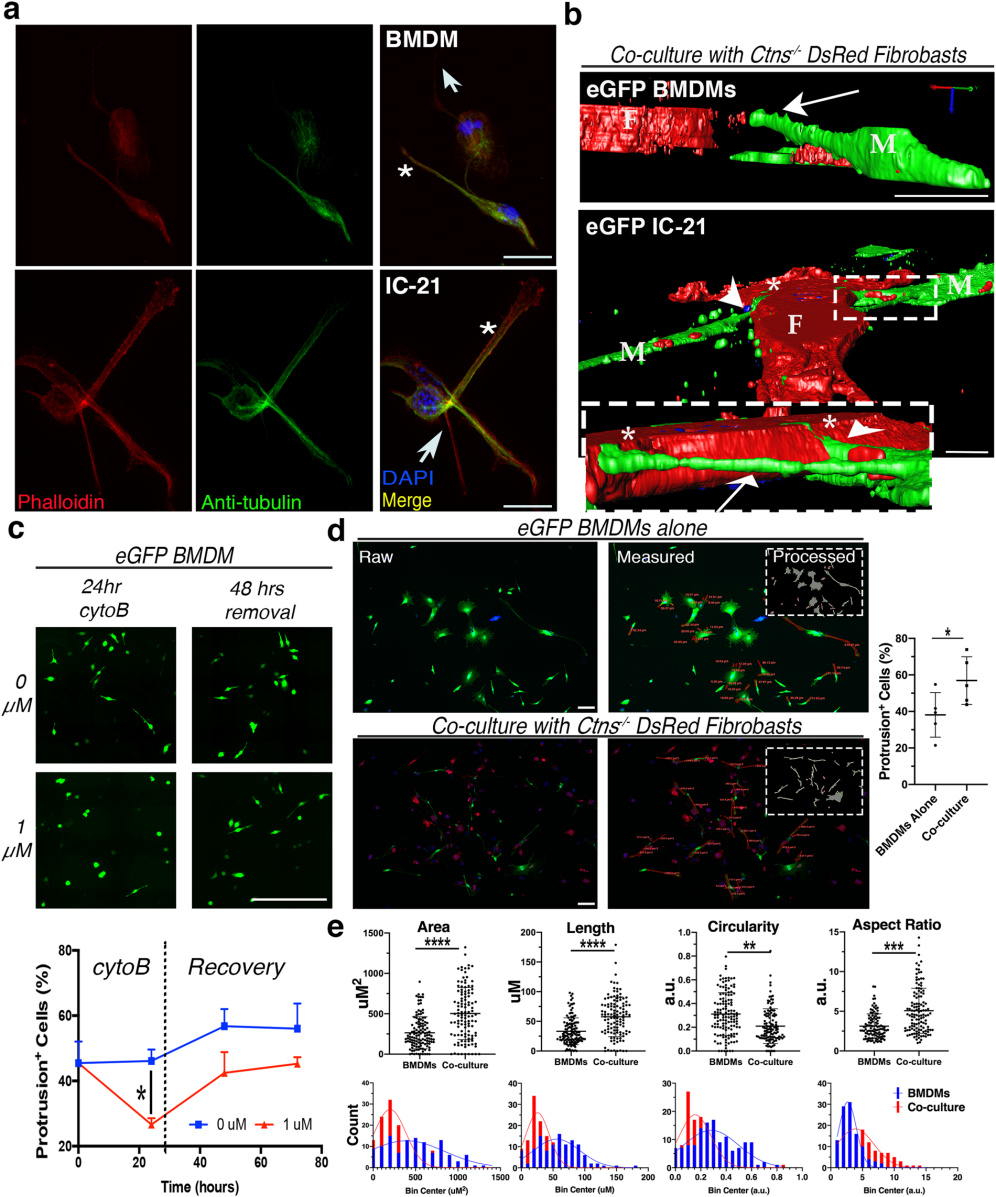
Some macrophage protrusions resemble TNT structure, behavior, and morphology. (**a**) Confocal micrographs of BMDMs and IC-21 cells after immunostaining for cytoskeletal components - actin (red) and tubulin (green). Thinner (arrows) and thicker (stars) protrusions indicated in both cell types. Scale bars: 10 µM. (**b**) 3D Z-stack reconstructions of eGFP^+^ BMDM or IC-21 cells (labeled M) co-cultured with *Ctns*^−/−^ DsRed^+^ fibroblasts (labeled F). Boxed insert highlights protrusions extending above dish surface (arrows) and resting along the substratum (arrowhead). See Supplementary Videos 1 and 2. Scale bars: 10 µM. (**c**) Representative stitched micrographs depicting eGFP^+^ BMDMs following treatment and removal of cytochalsin B (cytoB) compared to untreated controls. XY graph shows percentage of protrusion-positive cells in each condition over time (n = 3 regions per treatment). Scale bar: 500 µM. (**d**) Representative raw confocal micrographs and measured images for eGFP BMDMs alone and in co-culture with *Ctns*^−/−^ DsRed^+^ fibroblasts. Raw images were first processed for automated protrusion detection (insert) and the box-and-whisker plot (right) displays percentile quantification of protrusion-positive cells for BMDMs alone and in co-culture. Protrusion detection software rarely captures complete shape, so results were used as a guide in order to blindly manually separate protrusions from cell bodies by cutting along the estimation of normal membrane curvature. ImagePro was then used to measure protrusions; area measurements shown on top image and length on bottom. Scale bar: 100 µM. (**e**) Box-and-whisker plot (top) and frequency histogram (bottom) depicting area, length, circularity and aspect ratio protrusion measurements for BMDMs alone vs. in co-culture. Circularity is calculated as the ratio of the object compared to a circle with diameter equal to object’s maximum Feret diameter, and represented on a scale from 0–1.0, with 1.0 being a perfect circle. Aspect ratio is the ratio of the major and minor axis of an eclipse equivalent to the object, with a higher value meaning a more elongated object. Outliers identified and excluded by ROUT test. Curves fit to histograms by Gaussean linear regression. *P* values determined by one-way ANOVA or student’s *t*-tests. *P < 0.05, **P < 0.01, ***P < 0.001, ****P < 0.0001.

As no TNT-specific markers have been identified, we investigated the identity of macrophage-derived membrane protrusions based primarily on their morphology; TNTs are long, highly dynamic actin-rich tubes extending out from the plasma membrane without contacting the underlying substratum^33,34^. Cytoskeletal immunostaining of macrophage co-cultures revealed that all protrusions were actin-rich, while some also contained a tubulin core (Fig. 2a), a unique feature of thicker TNTs generated by macrophages mediating organelle transfer^26^. Protrusions were visualized in three dimensions via high-resolution imaging of Z-stacks and generation of 3D-reconstructions using ImagePro (Fig. 2b, Supplementary Videos 1 and 2). Both BMDMs and IC21 macrophages were found to have eGFP-positive protrusions extending above the substratum. Termini of the eGFP protrusions were observed directly adjacent to DsRed fibroblast cell bodies potentially indicating membrane fusion and facilitation of TNT stabilization through intercellular membrane connections. In addition, we treated BMDMs co-cultured with Ctns^−/−^ fibroblasts with cytochalasin B (cytoB), an actin destabilizing agent reported to prevent TNT formation and to inhibit organelle trafficking^35,36^. Treatment with cytoB for 24 hours eliminated membrane protrusions from BMDMs with the extensions being restored several days following removal of cytoB (Fig. 2c).

In order to further characterize protrusions, we analyzed the shape and size of the automatically detected regions. We seeded and imaged equal numbers of eGFP BMDMs either alone or in co-culture with DsRed *Ctns*^−/−^ fibroblasts. We first used our image analysis workflow to determine that co-cultures had a higher percentage of cells with protrusions than BMDMs alone (*P* = 0.046) (Fig. 2d). Using the automatically detected regions as guides, we blindly separated complete protrusions from the cell body, then used ImagePro to measure the resultant regions. From both conditions combined, we measured 242 individual protrusions and found that the mean protrusion area was 380.5 ± 145.4 µm^2^ and mean length was 45.00 ± 15.41 µm. As TNTs are extremely diverse, there is considerable variation on what “standard” dimensions between cell types^37^. That said, most authors report an average TNT length between 17.7 µm (Jurkat T cells) and 44 µm (ARPE-19 retinal pigment epithelial cells), while the longest protrusions can reach up to 120 µm, confirming our measurements are compatible with TNTs^34,38^. Unexpectedly, we also determined that protrusions generated from co-cultured macrophages were larger, longer and more elongated, meaning that they were less circular and had a larger ratio of the major to minor axis (Fig. 2e). These data not only confirm that a sizable fraction of protrusions appear to be of appropriate size to be considered TNTs but also highlights how the presence of diseased cystinotic cells affects protrusion morphology, as well as frequency. This is particularly interesting considering that previous work has shown that thicker macrophage TNTs preferentially transport organelles^26^.

Finally, we analyzed expression of several genes involved in TNT formation following macrophage polarization to assess any differential expression of TNT-associated genes (Supplementary Fig. S4). In M(LPS/IFNγ) BMDMs, we observe increased mRNA expression of the MHC class II protein leukocyte specific transcript 1 (*Lst1)*. In contrast, M(LPS/IFNγ) BMDMs displayed decreased expression of the Rho-family GTPase cell division control protein 42 homolog (*Cdc42*). We did not detect any changes in other TNT-linked genes such as *M-Sec*, a component of the exocyst complex that directly promotes TNT formation nor did we detect any changes in protein expression (data not shown)^39^. Such changes in TNT marker expression after polarization are intriguing but thorough study of protein activity and localization would be required to uncover any mechanistic links between polarization and TNT formation. As such, in terms of protrusion characterization, a focus on structure, behavior and morphology is the more reliable method to indicate that a good fraction of the macrophage-derived protrusions automatically observed in our co-culture conditions represent *bona fide* TNTs.

### Co-culture of macrophages with cystinotic cells increased not only lysosomal but mitochondrial intercellular transfer as well

To investigate the potential impact of macrophage polarization on TNT-mediated intercellular organelle trafficking, *Ctns*^−/−^ and WT DsRed^+^ fibroblasts were co-cultured with IC-21 macrophages stably transduced with the fusion proteins cystinosin-eGFP or frataxin-eGFP, a mitochondrial iron chelator defective in Friedreich’s ataxia^40^. *In vitro* live imaging previously revealed transfer of cystinosin-eGFP-bearing lysosomes or frataxin-eGFP-bearing mitochondria from macrophages to diseased murine *Ctns*^−/−^ or *Fxn*^−/−^ fibroblasts, respectively^7,8^. High-magnification 3D-reconstructions confirmed the presence of eGFP-positive organelles within the cytoplasm of DsRed^+^ fibroblasts, as demonstrated by 3D renderings and orthogonal projections (Supplementary Fig. S5a,b, Supplementary Videos 3 and 4). Lysosomal transfer was confirmed to occur through TNT-like structures (Supplementary Fig. S5c, Video 5).

**Figure 3:**
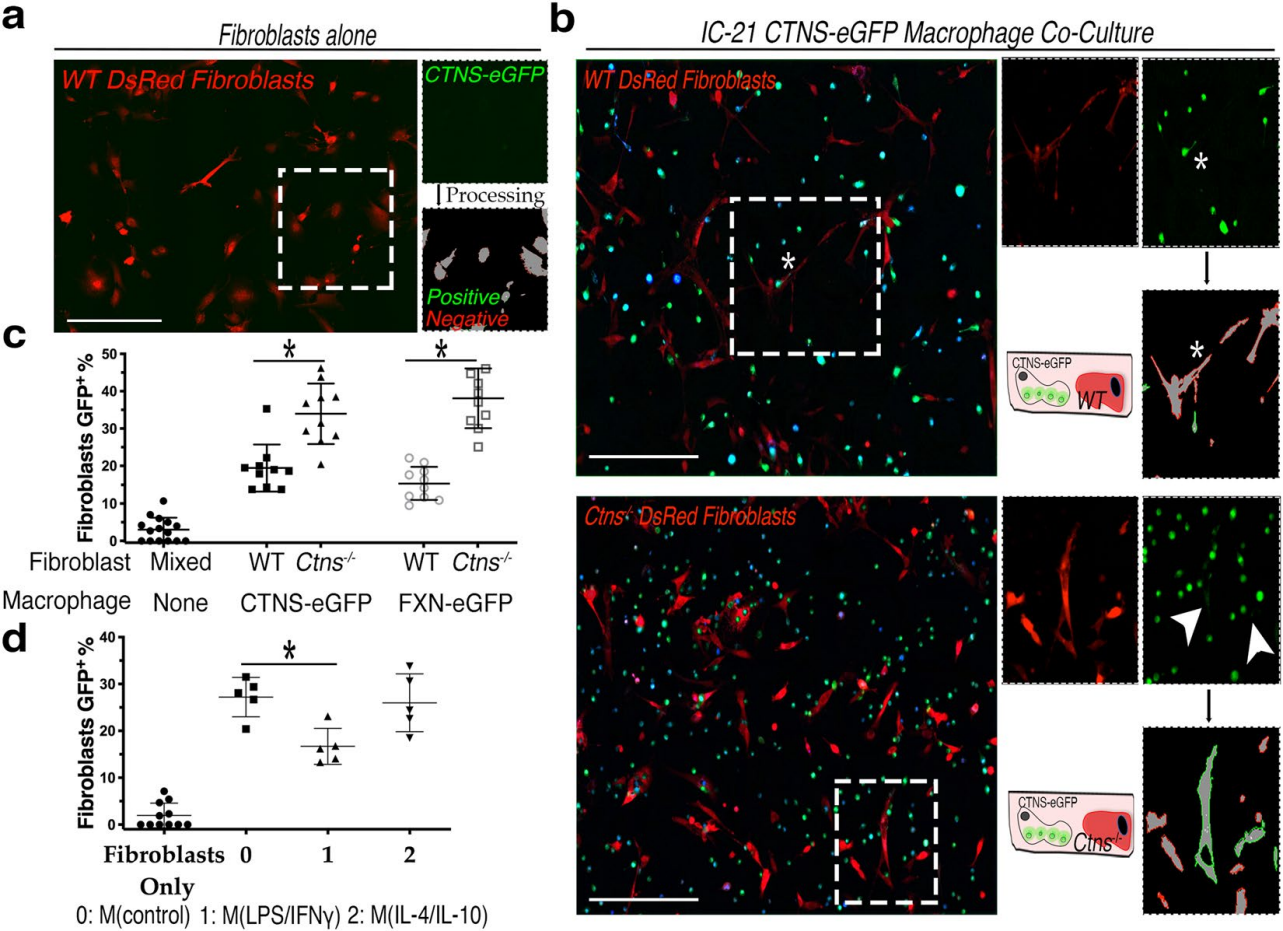
Intercellular trafficking of cystinosin-eGFP lysosomes and frataxin-eGFP mitochondria is increased to diseased *Ctns*^−/−^ fibroblasts and diminished following proinflammatory polarization of co-cultured macrophages. (**a**) Representative stitched fluorescent micrograph of essential co-culture background control with only recipient DsRed^+^ fibroblasts. Boxed insert depicts automated image processing showing donor-positive (green) and donor-negative cells (red). See Supplementary Fig. S3 for automation methodology. (**b**) Representative fluorescent images of IC-21 cystinosin-eGFP co-cultures with either WT or *Ctns*^−/−^ DsRed^+^ fibroblasts. Zoomed inserts highlight automated transfer quantification. Arrowheads indicate eGFP signal within DsRed^+^ fibroblasts. Stars indicate excluded signal due to cellular overlap. (**c**) Percentile quantitation of cystinosin- or frataxin-eGFP^+^ fibroblasts via automated image analysis. Each data point represents percent of donor-positive recipient cells in one stitched image (n = 3). All co-culture conditions have significantly more donor eGFP signal than background control with only fibroblasts (*P* < 0.05). (**d**) Frequency of cystinosin-eGFP trafficking in macrophage and fibroblast co-cultures following polarization with comparison to fibroblasts alone control. Histogram showing percentage of fibroblasts automatically determined positive for eGFP signal (n = 5). All scale bars are 500 µM. All graphs shown as mean ± SD. *P* values determined by one-way ANOVA or student’s *t*-tests. *P < 0.05, **P < 0.01.

We quantified the efficiency of lysosomal and mitochondrial transfer from the macrophages to the fibroblasts using another image analysis algorithm similar to the one we developed for TNT quantification, which measures the frequency of eGFP puncta (“donor”) within a region of interest (ROI) defined by DsRed^+^ “recipient” cell (Supplementary Fig. S6). Co-cultures of 75,000 cystinosin-eGFP or frataxin-eGFP-expressing IC-21 macrophages and 50,000 DsRed^+^ WT or *Ctns*^−/−^ fibroblasts were grown for three days and then fixed; fibroblasts cultured alone were used as controls (Fig. 3a). Fluorescent imaging revealed significantly higher numbers of both cystinosin-eGFP lysosomes (*P* = 0.042) and frataxin-eGFP mitochondria (*P* = 0.019) in *Ctns*^−/−^ fibroblasts compared to WT fibroblasts (Fig. 3b,c, Supplementary Fig. S6c). Further, we treated macrophages for co-culture experiments to determine if either macrophage subtype impacts the efficacy of intercellular trafficking. We observed a significantly decreased presence of cystinosin-eGFP-positive lysosomes within fibroblasts co-cultured with M(LPS/IFNγ) macrophages (*P* = 0.045) (Fig. 3d).

**Figure 4:**
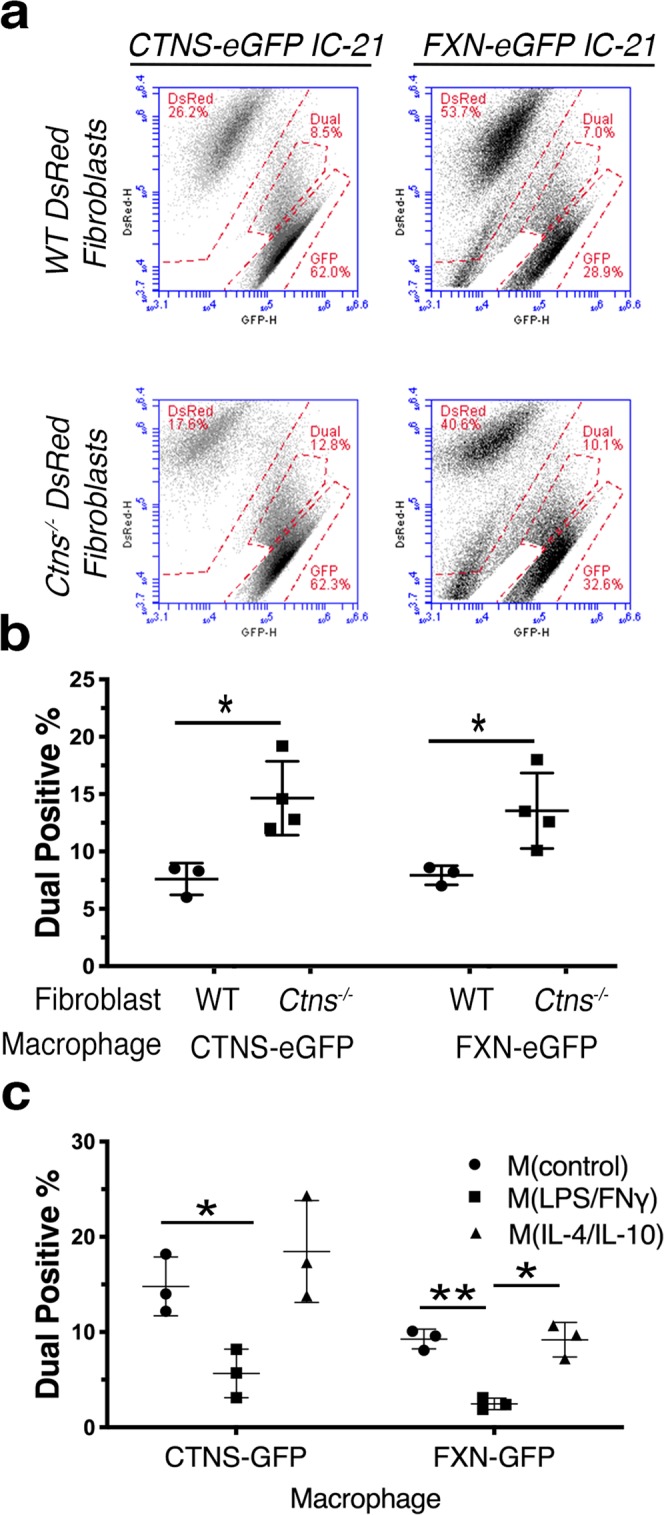
Flow cytometry confirms that intercellular trafficking is increased to diseased *Ctns*^−/−^ fibroblasts and suppressed by proinflammatory polarization. (**a**) Representative FACS plots of DsRed (y-axis) and eGFP (x-axis) for 50,000 cells following co-culture of either cystinosin- or frataxin-eGFP and WT or *Ctns*^−/−^ DsRed^+^ fibroblasts. Signal between eGFP and DsRed gates classified as a “dual-positive” eGFP^+^DsRed^+^ population. See Supplementary Fig. S4 for sorting and gating strategy. (**b**) Percentage quantification of eGFP^+^DsRed^+^ dual-positive cells in cystinosin- or frataxin-eGFP macrophage co-cultures with WT or *Ctns*^−/−^ DsRed^+^ fibroblasts. Replicates consisted of co-cultures with fibroblasts derived from multiple mice (n = 4). (**c**) Quantification of dual-positive cell frequency after polarization treatment of co-cultures with cystinosin- or frataxin-eGFP and *Ctns*^−/−^ DsRed^+^ fibroblasts (n = 3). All graphs shown as mean ± SD. *P* values determined by one-way ANOVA or student’s *t*-tests. *P < 0.05, **P < 0.01.

To corroborate our findings using another robust assay, we performed flow cytometry on co-cultures to analyze dual eGFP- and DsRed-positive cells; both cell types cultured alone were used as gating controls (Fig. 4a, Supplementary Fig. S7a). Consistent with our fluorescent imaging results, a significant increase of approximately 70–90% of the dual-positive population was observed in co-cultures of *Ctns*^−/−^ fibroblasts with both cystinosin-eGFP (*P* = 0.0168) or frataxin-eGFP (*P* = 0.0371) macrophages as compared to WT fibroblast co-cultures (Fig. 4b). Another control of eGFP^+^ and DsRed^+^ cells mixed immediately prior to sorting did not yield dual-positive cells, indicating that prolonged co-culture is necessary to facilitate cellular interaction resulting in the generation of an eGFP^+^DsRed^+^ population (Supplementary Fig. S7b). Furthermore, proinflammatory macrophage polarization also dramatically decreased the transfer of cystinosin-eGFP and frataxin-eGFP (Fig. 4c). Altogether, these data show that diseased cells enhance transfer of lysosomes and mitochondria from macrophages to the fibroblasts, while proinflammatory polarization inhibits this process.

### HSPC-derived macrophages within the kidney of transplanted *Ctns*^−/−^ mice appear to preferentially express M(LPS/IFNγ) markers

As *in vitro* polarization indicates that proinflammatory M(LPS/IFNγ) macrophages have impaired TNT formation and organelle transfer, we assessed the *in vivo* macrophage phenotype following HSPC transplantation in *Ctns*^−/−^ mice. We transplanted WT eGFP HSPCs into *Ctns*^−/−^ recipient mice via tail vein injection as previously described^14^ and analyzed macrophage polarization markers by performing immunostaining on kidney sections (Supplementary Fig. S8). We observed robust colocalization between the eGFP^+^ HSPC progeny and the pan-macrophage marker CD68 (Supplementary Fig S8a). Unexpectedly, more abundant co-localization was detected between the stem cell progeny and markers for M(LPS/IFNγ) such as CD16/32 or MHCII, than with anti-inflammatory markers ARG1, CD206 or CD163 (Supplementary Fig. S8b). Antibodies were tested on other tissues such as tumors to ascertain positive immunoreactivity (Supplementary Fig. S8c). These data suggest that *in vivo* proinflammatory macrophages are responsible for tissue preservation after WT HSPC transplantation in cystinosis.

### Disruption of anti-inflammatory macrophage polarization with *Rac2*^−/−^ HSPCs has no effect on HSPC transplantation efficacy or TNT formation

To determine if proinflammatory macrophages are responsible for tissue preservation in cystinosis following HSPC transplantation, we genetically perturbed macrophage polarization to assess if deficiencies prevented rescue of the cystinosis phenotype following HSPC transplantation. The *Rac2*^−/−^ mouse model, disrupting the hematopoietic-specific Rho family GTPase *Rac2*, was chosen due to its functions in actin cytoskeletal reorganization and macrophage polarization^41,42^. Since RAC2 was shown to drive M(IL-4/IL-10) macrophage differentiation, the proinflammatory M(LPS/IFNγ) macrophage population was enriched in knockout *Rac2*^−/−^ mice^43–45^. A very similar family member, RAC1, also has a role in the biogenesis of macrophage protrusions including invadopodia, lamellipodia and TNTs^42,46^.

**Figure 5:**
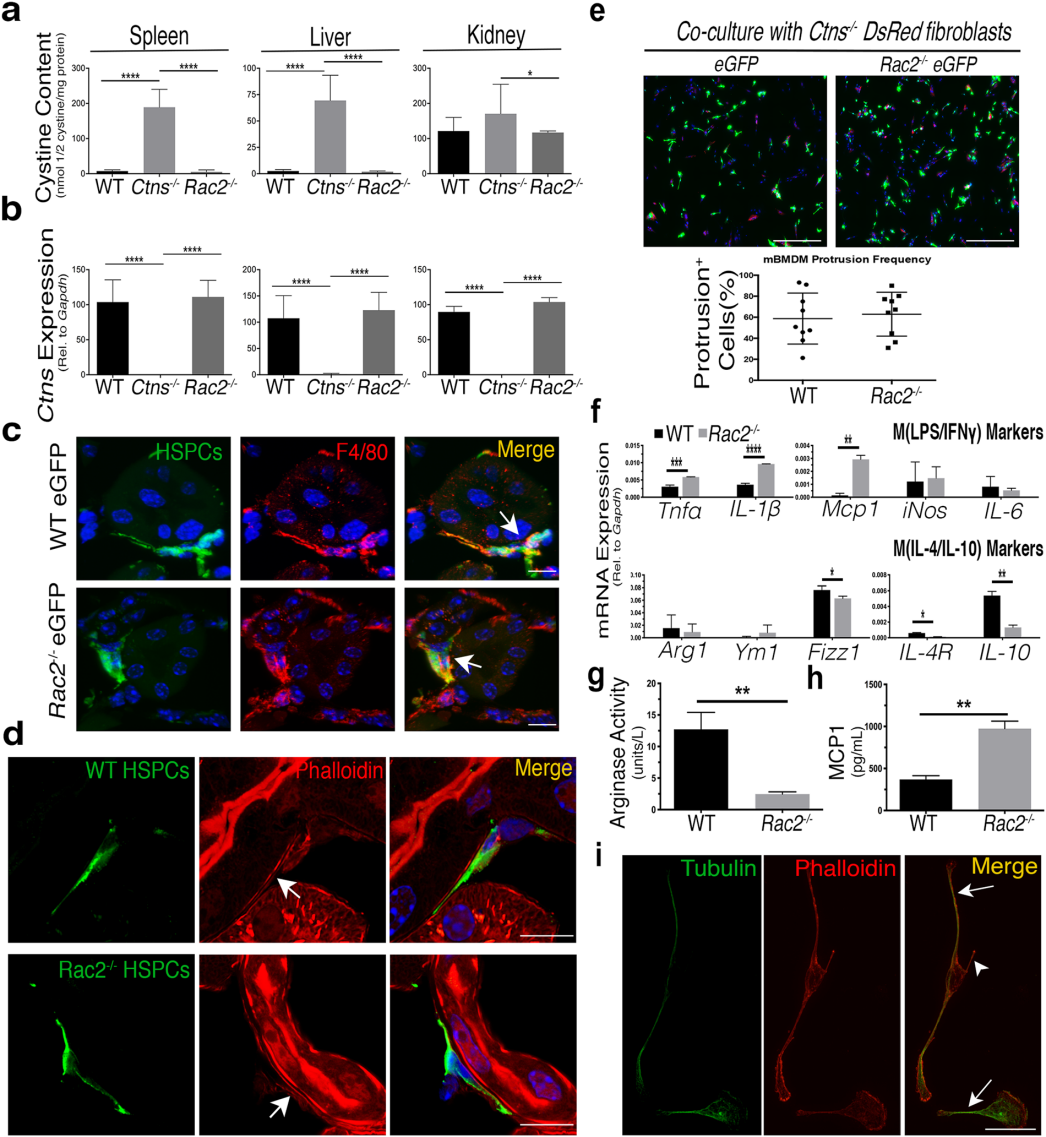
Enrichment of proinflammatory macrophages *in vivo* by transplantation of *Rac2*^−/−^ HSPCs has no effect on efficacy in *Ctns*^−/−^ mice or on BMDM TNT formation *in vitro*. (**a**) Histograms representing cystine content in spleens, livers and female kidneys as measured by mass spectrometry in *Ctns*^−/−^ mice transplanted with WT HSPCs (n = 5), *Ctns*^−/−^ HSPCs (n = 4), and *Rac2*^−/−^ HSPCs (n = 4). Only female kidneys are shown, as previous observations indicate that kidneys need to be analyzed by sex^16^, and only 2 males were in the smallest recipient group. (**b**) mRNA quantitation of murine *Ctns* expression of transplanted *Ctns*^−/−^ mice in spleen, liver and kidney. Histograms represent changes in *Ctns* expression relative to housekeeping control *Gapdh*. (**c**) Representative confocal micrograph of kidney sections of *Ctns*^−/−^ mice transplanted with either WT or *Rac2*^−/−^ HSPCs (green), probed with the macrophage marker F4/80 (red) and the nuclear stain DAPI (blue). Arrows indicate colocalization between eGFP HSPC progeny and F4/80 expression. (**d**) Confocal micrograph depicting kidney sections of a *Ctns*^−/−^ mouse transplanted with WT (top) or *Rac2*^−/−^ (bottom) eGFP HSPCs reveals thin eGFP TNT-like protrusions extending away from the cell body. Frozen sections were stained with anti-GFP to enhance visibility of HSPC progeny, and then probed with rhodamine phallodin to highlight actin within TNT-like structures (arrows). Scale bars: 10 µM. (**e**) BMDMs isolated from WT or *Rac2*^−/−^ bone marrow were co-cultured with *Ctns*^−/−^ DsRed^+^ fibroblasts and assesed for frequency of protrusions using automated analysis software (see Supplementary Fig. S1). Histogram displays quantification of percentage of BMDMs positive for protrusions (n = 3). Scale bar: 500 µM. (**f**) Bar graph displays mRNA expression of each individual pro- and anti-inflammatory macrophage polarization gene relative to housekeeping control *Gapdh* in WT or *Rac2*^−/−^ BMDMs (n = 3). (**g**) Bar graph shows measurement of proinflammatory MCP1 cytokine secretion in conditioned media derived from WT or *Rac2*^−/−^ BMDMs assessed using ELISA (n = 3). (**h**) Bar graph represents colorimetric measurements of M(IL-4/IL-10) marker ARG1 enzymatic activity in WT or *Rac2*^−/−^ BMDMs normalized by comparison to blank with a standard curve (n = 3). (**i**) Confocal micrograph of *Rac2*^−/−^ BMDM cytoskeletal immunostaining of tubulin (green) and actin (red) reveals some *Rac2*^−/−^ BMDM protrusions only have actin (arrowhead) with others also containing tubulin (arrows). All scale bars are 10 µM except otherwise noted. All graphs shown as mean ± SD. *P* values were determined by one-way ANOVA or students *t*-tests. *P < 0.05, **P < 0.01, ***P < 0.001, ****P < 0.0001.

To test if *Rac2* elimination has any effect on HSPC transplantation efficacy, HSPCs isolated from *Rac2*^−/−^ eGFP-transgenic mice were transplanted into 2-month-old *Ctns*^−/−^ recipient mice. *Ctns*^−/−^ mice transplanted with WT eGFP^+^ or *Ctns*^−/−^ eGFP^+^ HSPCs proven effective or ineffective at prevention of cystinosis, respectively, were used as controls^14^. HSPCs engraftment was measured by quantifying eGFP^+^ peripheral blood cells, which did not significantly differ between the three transplanted groups (average for WT −71.5%, *Ctns*^*−/−*^ −75.6% and *Rac2*^−/−^ −65.9%). Mice were sacrificed six months post-transplantation, and samples were collected for biochemical and phenotypic characterization. Assessment of tissue cystine content in WT HSPC recipients resulted in a dramatic reduction in cystine load in the liver, spleen, and kidneys relative to *Ctns*^−/−^ HSPC-transplanted animals, as expected (Fig. 5a). Surprisingly, transplanted *Rac2*^−/−^ HSPCs resulted in a decrease of cystine content to the same magnitude as WT HSPC controls. In addition, rescue of *Ctns* expression in tissues was similar in WT and *Rac2*^−/−^ HSPC-transplanted *Ctns*^−/−^ mice (Fig. 5b). Taken together, these data indicate that *Rac2*^−/−^ HSPC transplantation is fully capable of restoring functional cystinosin to diseased tissue.

We then analyzed kidney sections by immunofluorescence for immune cell infiltration and observed co-localization between *Rac2*^−/−^ eGFP HSPC progeny and macrophage markers such as F4/80 or CD68 (Fig. 5c), indicating that typical macrophage differentiation remained unaltered. We also detected eGFP TNT-like protrusions within kidneys of both *Rac2*^−/−^ and WT HSPC recipients that stained positive for actin, another hallmark of TNTs (Fig. 5d). These data confirm that knockout HSPC-derived cells retain the ability to form TNT-like protrusions *in vivo*.

Finally, we investigated the protrusive behavior and polarization phenotype of BMDMs isolated from *Rac2*^−/−^ eGFP mice *in vitro*. We quantified co-cultures of freshly isolated WT eGFP^+^ and *Rac2*^−/−^ eGFP^+^ BMDMs with *Ctns*^−/−^ fibroblasts using our protrusion detection imaging algorithm (Supplementary Fig. S1) and observed no significant differences in protrusion frequency between WT- and *Rac2*^−/−^- BMDMs (*P* = 0.0472; Fig. 5e). We then analyzed polarization markers and confirmed that in contrast with WT BMDMs, *Rac2*^−/−^ BMDMs skew towards M(LPS/IFNγ) without any extrinsic stimulation (Fig. 5f–h). However, we note that the magnitude of change in mRNA expression of genes was overall either less dramatic (*Tnfα*, *Il-10* or *Fizz1)* than the M(LPS/IFNγ) macrophages treatment *in vitro* (Fig. 1a) or were not significantly different between genotypes (*iNos*, *Arg1* or *Il-6)* (Fig. 5f). Even so, a robust increase in MCP1 secretion and significantly decreased ARG1 enzymatic activity compared to WT BMDMs further confirmed the proinflammatory phenotype of the *Rac2*^−/−^ macrophages (Fig. 5g,h). However, *Rac2*^−/−^ eGFP BMDMs still formed tubes resembling TNTs consisting of actin and tubulin cytoskeletons (Fig. 5i) found lying above the substratum surface (data not shown). Taken together, these results indicate that while the polarization phenotype of *Rac2*^−/−^ eGFP^+^ BMDMs is skewed towards a M(LPS/IFNγ) phenotype, both formation of TNT-like protrusions and *Rac2*^−/−^ HSPCs transplantation efficacy remain unaffected.

## Discussion

In the present study, we investigated the phenotype of the HSPC-derived macrophages responsible for intercellular trafficking through TNTs. Several decades of research into macrophage subpopulations has elucidated differences in cytokine production, glucose metabolism, secondary messengers, stimulatory conditions, cellular morphology and numerous other factors which collectively lead to differences in macrophage biological activity, potentially including TNT-mediated trafficking^27,28^. Using established assays in conjunction with novel imaging workflows, we observed a reduction in TNT formation and organelle transfer following LPS/IFNγ polarization, suggesting optimal TNT trafficking is impaired with increased proinflammatory stimulation. Unlike other imaging analysis platforms, our protrusion detection methodology requires no antibody staining or fixation and can function at relatively low magnification (40x), enabling large numbers of cells to be quickly and automatically analyzed without bias^47,48^. Development of these tools not only increased our understanding of the morphological dynamics of macrophage polarization but also highlighted the potential for further study of cellular protrusion dynamics. This ImagePro Premier workflow could potentially allow quantitative analysis of other fluorescently-labeled cellular extensions such as filopodia, neuronal axons, or invadopodia in cancer.

Following HSPC transplantation *in vivo*, tissue-integrated macrophages appeared to adopt a predominantly proinflammatory phenotype (Supplementary Fig. S8). In addition, HSPCs isolated from the *Rac2*^−/−^ mouse model were enriched for a M(LPS/IFNγ) macrophage population but appeared to be as efficient in rescuing cystine build-up in the *Ctns*^−/−^ mice as WT HSPCs. Our findings correlate with a recent study that demonstrated increased recruitment of proinflammatory immune cells in cystinosis, further highlighting the crucial role that inflammation plays in this disorder^49^. In addition, the dissonance between our *in vitro* and *in vivo* results reflects mounting evidence that the *in vitro* paradigm of macrophage activity can be a potentially misleading oversimplification^25^. Further studies applying similar methodology to more complex *in vitro* models such as 3D culturing or iPSC-derived organoids or spheroids may help to bridge the gap between findings from cell culture and mouse models. That said, *in vitro* polarization can easily push cells beyond physiological levels of polarization. For example, the mRNA expression of important inflammatory mediators, including the master cytokine regulator *TNFα*, increased in M(LPS/IFNγ) IC-21 macrophages by over an order of magnitude; however, the magnitude of increase between *Rac2*^−/−^ and WT BMDMs was far more modest. In terms of TNT-like protrusion formation, the reduced disruption of polarization induced by lack of RAC2 might be mitigated by redundant pathways or signaling. Alternatively, these results could show that RAC2 itself has no role in TNT formation. In contrast, siRNA-mediated knockdown of RAC1 results in a reduction of TNTs^50^. Although the two proteins are quite similar (92%), Miskolci *et al*. report substantial differences in their subcellular localization – upon stimulation, RAC1 is primarily localized to the periphery, while RAC2 was distributed throughout the cell^51,52^. Altogether, these data reveal that induction and function of TNT-like protrusions can be achieved by macrophages across the physiological spectrum of polarization, but is impaired when exogenous stimulation pushes cells to an extreme physiological state.

Like all cellular protrusions, TNTs require extensive modification and reorganization of the cytoskeleton to distort the plasma membrane and generate the protrusive force necessary to reshape the cell, elongate, and dynamically maintain the protrusion once formed^1^. Evidence also strongly implicates macrophage polarization in differentially modulating cytoskeletal dynamics and reorganization^53,54^. These shared pathways suggest polarization-induced alterations in expression and/or activity of TNT-regulator proteins could be responsible for the reduction in TNT-like protrusion formation following proinflammatory stimulation. We observed a reduction in M(LPS/IFNγ) BMDM mRNA expression of the known TNT inducer *Lst1* which acts as a scaffold to recruit many proteins including filamin and the small GTPase RalA to the plasma membrane in order to generate and maintain TNTs^55^ (Supplementary Fig. S4). We also observed that proinflammatory BMDMs have decreased mRNA expression of *Cdc42*, a Rho-family GTPase involved in macrophage polarization which binds N-WASP to control actin branching through Arp2/3^42,56^. In macrophages along with Jurkat and HeLa cells, inhibition of *Cdc42* reduces TNT formation and intercellular trafficking^39,50,57^. Interestingly, in neurons *Cdc42* actually negatively regulates TNTs and trafficking while promoting filopodial formation^51^. While one can speculate that the reduction of M(LPS/IFNγ) protrusion formation could be due to the increase of *Lst1* disrupting normal formation of membrane focal points and/or because of the decrease of *Cdc42* actin branching activity, drawing such mechanistic conclusions requires far more evidence than mRNA marker expression. That said, even though a direct connection between these expression changes and TNTs remain unclear, our data does support the central role of actin regulating Rho-family GTPases in TNT formation and provides further evidence of their involvement in macrophage polarization.

A major functional consequence of proinflammatory macrophage polarization was also a clear reduction in intercellular trafficking following induction of this phenotype (Fig. 3). Macrophages specifically are known to frequently form TNTs that are “thicker” and contain a tubulin core in addition to actin, which are known to be responsible for organelle trafficking^7,26^. Correspondingly, we observed an increase in protrusion frequency, size, length and elongated shape when BMDMs were cultured with cystinotic cells compared to macrophages alone (Fig. 2d,e). This observation combined with the shared reduction of trafficking and TNT-like protrusion formation following *in vitro* LPS/IFNγ polarization strongly supports the idea that TNTs form a major artery of intercellular delivery of lysosomes and mitochondria. Interestingly, not only were more cystinosin-eGFP-bearing lysosomes delivered to the diseased cells, which lack the lysosomal transmembrane protein cystinosin, but an increase in transfer of frataxin-eGFP-bearing mitochondria was also observed to cystinotic cells. Two hypotheses could explain these findings: (i) TNTs facilitate widespread delivery of cellular organelles to diseased cells so the fact that there are more thick TNTs inevitably leads to more trafficking of all cargos. No known TNT “gating” mechanisms have been identified to disprove this hypothesis, although overall protrusion structure has been shown to affect the types of molecular cargos transferred^26^; (ii) Cystinosis also causes mitochondrial dysfunction characterized by dysregulation of the cyclic AMP cycle and impaired autophagy^58,59^. Therefore, it is also possible that whatever unknown media-soluble factor(s) causing increased TNT-like protrusion formation and thicker morphology also stimulates transfer of mitochondria as well as lysosomes. Whatever the cause may ultimately be, the finding that diseased cells increased macrophage-derived thick TNT formation and intercellular trafficking activity represents exciting new possibilities for the study of TNTs. A major thread of research from numerous groups has focused on how TNTs are involved in the transfer of harmful pathogenic molecules – ranging from HIV infection in T cells to cancer cell invasion to spreading misfolded tau protein aggregates^17,34,60^. Thus, our findings that diseased cystinotic cells cause macrophages to increase TNT-mediated trafficking highlights the idea of TNTs as delivery system for regenerative medicine as opposed to mediating the spread of deleterious agents.

In this study, we investigated the phenotype of macrophages responsible for the widespread dissemination of molecular cargo via TNTs both *in vitro* and *in vivo*. We developed novel imaging tools allowing for automatic and unbiased quantification of cellular protrusions as well as intercellular trafficking efficacy in order to investigate these behaviors in the context of polarized macrophages. Given the apparent widespread and versatile nature of macrophage TNT transport, it is likely that many molecular products beyond functional lysosomes or mitochondrial proteins are transferred via TNTs; cargos that may not be readily secreted and thus be more difficult to deliver widely. Thus, understanding the mechanisms and cellular type that are responsible for TNT-mediated delivery will enhance the efficacy of numerous potential *ex vivo* therapeutic approaches targeting a wide variety of loss-of-function organelle disorders.

## Methods

### Mice and ethics statement

C57BL/6 *Rac2*^−/−^ mice were provided by Dr. Durden (Moores Cancer Center, University of California, San Diego) and C57BL/6 *Ctns*^−/−^ mice were provided by Dr. Antignac (Inserm U983, Paris, France). Enhanced green fluorescent protein (eGFP; C57BL/6-Tg(ACTB-EGFP)1Osb/j) and DsRed (B6.Cg-Tg(CAG-DsRed*MST)1Nagy/J) transgenic mice were purchased from The Jackson Laboratory (Bar Harbor, ME). *Ctns*^−/−^ mice were cross-bred to both DsRed and GFP to produce *Ctns*^−/−^ strains constitutively expressing either DsRed or GFP reporter genes as previously described^14,16^. All strains and mouse procedures were approved by the University of California, San Diego (UCSD) in accordance with the guidelines set forth by the Institutional Animal Care and Use Committee (Protocol ID S12288).

### Macrophage and fibroblast isolation and culture

BMDMs were isolated from C57BL/6 mice according to standard protocols as described previously^61^. Briefly, mice were sacrificed using isoflurane, femurs and tibias were removed, and bone marrow was flushed using PBS with 1% fetal bovine serum (FBS). Cells were cultured at 37 °C and 5% CO_2_ in complete RPMI-1640 media (10% FBS, 2% penicillin/streptomycin) supplemented with 20% mCSF-containing L929-conditioned medium for seven days on tissue-culture (TC) treated plastic, with a media change at day 3. BMDMs were grown for no more than three passages and subcultured by scraping. For *in vitro* polarization experiments, BMDMs or IC-21 were cultured for at least 48 hours in complete media with either 100 ng/mL LPS and 50 ng/mL IFNγ or 10 ng/mL IL-4 and IL-10 **(**BioLegend**)** for M(LPS/IFNγ) and M(IL-4/IL-10) polarization, respectively. Primary fibroblasts were isolated from neonatal mouse skin biopsies by allowing them to grow out of small skin pieces (~0.5 cm^2^) attached to the culture dishes for several days prior to skin removal and subculture in complete DMEM media (10% FBS, 2% penicillin/streptomycin).

### Co-culture growth and imaging

75,000 macrophages and 50,000 fibroblasts were cultured together in 6-well dishes under polarization conditions for two days. Cells were washed with warm PBS and fixed for 10 mins at 37 °C in 4% paraformaldehyde (PFA). Cells were washed in PBS again and imaged at 40X on the BZX-700 Fluorescent Microscope (Keyence). Five large image stitches (5 × 5) were generated per experimental condition, with acquisition settings held constant within experimental runs.

### Imaging analysis: protrusion quantification

Stitched images were calibrated in ImagePro Premier (Media Cybernetics) for automated quantification of protrusion frequency. eGFP^+^ macrophages were size-selected by filtration of objects with an area 100 µm^2^ or larger and then masked to reduce noise artifacts (Supplementary Fig. S1a, Step I). Protrusions were isolated by repeatedly passing a morphological erosion filter (2 × 2 square, 7 passes) over the image which erased peripheral cellular signal, followed by applying a dilation that restored signal to the central cell body (Step II). Subtracting the processed image from the original mask resulted in selection of regions protruding from the main cell body, which can be filtered by a highly variable set of size parameters to yield either thin TNT-like structures or other thicker protrusions (Supplementary Fig. S1b) (filters for TNTs: 20 µm^2^ minimum area, 10 µm^2^ minimum longest axis, 0.6 maximum circularity, 1.9 box area minimum). Finally, the selected protrusion area was overlaid with the original image and cells were classified as either protrusion-positive or -negative based on signal intensity; overlap of protrusion and cell body yielded a positive result (Step III - IV). All segmentation, adjustment and filtration factors were trained and optimized blind to condition and applied automatically to batch images using ImagePro Premier macros.

### Imaging analysis: transfer quantification

Stitched images were imported as above and split into “donor” and “recipient” (DsRed) channels. Donors included genetically-encoded, fluorescently-labeled organelles like cystinosin-eGFP lysosomes and frataxin-eGFP mitochondrial protein, as well as transient stains (for example, Wheat Germ Agglutin (WGA) labels of the plasma membrane). Recipient cell outline was selected (minimum area 100 µm^2^) and applied as a region of interest (ROI) to the donor channel. Any signal within this ROI was adjusted and selected, taking caution to use controls lacking any donor signal to establish fluorescent background (Supplementary Fig. S3b, top). Filtered donor signal was combined with original recipient outline and signal overlap was used again to classify recipients as donor-positive or -negative. Exclusion of any signal at the boundary of recipient cells, as well as size filtration of donor molecules (maximum area 25 µm^2^), helped reduce false-positive imaging artifacts.

### Flow cytometry

Co-cultures were seeded as described previously, but onto non-tissue culture-treated plastics to enable trypsinization of macrophages. Co-cultures were washed in ice-cold PBS, trypsinized at room temperature (RT) and rapidly rinsed in cold media before centrifugation at 4 °C. Cells were resuspended in cold PBS and vortexed, then run through the BD Accuri C6 cytometer (BD Biosciences). Debris was filtered by SSC/FSC gating with 50,000 cellular events collected. Macrophages and fibroblasts without any fluorescent tags were used to establish an exclusion region from final co-culture sort, while eGFP^+^, DsRed^+^ and dual-positive gates were drawn with each cell type seeded alone. For macrophage polarization markers, BMDMs were polarized as described, then scraped and resuspended in ice cold FACS buffer (1% FBS, 0.1% sodium azide in PBS). Cells were treated with Fc block (BD Biosciences) for 10 mins before incubation for 1 hr on ice in the dark with appropriate antibodies. Cells were washed three times in FACS buffer before immediately reading on the cytometer. Antibodies are as follows: F4/80 PE (Biolegend 565410), CD80 FITC (Biolegend 104706), CD126/IL6Rα APC (115811), CD206 PE (Biolegend 141795).

### Quantitative PCR

To measure gene expression in BMDMs/IC-21 and explanted tissues, total RNA was extracted with TRIzol Reagent (Invitrogen) per manufacturer’s recommendations. A total of 1 μg isolated RNA was reverse transcribed into cDNA with the iScript cDNA synthesis kit (Bio-Rad). Real-time PCR was performed using iTaq Universal SYBR Green on a CFX96 thermocycler (Bio-Rad) under the following conditions: 95 °C (30 s), 40 cycles of 95 °C (5 s) and 60 °C (30 s), then 65 °C (5 s) and finally 95 °C (5 s). Reaction mixture consisted of 3 μL cDNA (5 ng/ul), 0.6 μL forward and reverse primer (10 μM), 1.8 μL H20 and 5 μL SYBR green. Samples were normalized to Glyceraldehyde 3-phosphate dehydrogenase (*Gapdh*) and analyzed according to the delta/delta Ct method. All primer sequences are shown in Supplementary Table S1.

### Immunoblotting

Total protein was harvested and resuspended in 1% SDS from cultures of 1 × 10^6^ BMDMs and IC-21 using TRIzol Reagent (Invitrogen) per manufacturers protocol and quantified using the Pierce BCA Protein Assay kit (Thermo Fisher Scientific). 10–20 μg of protein was subjected to SDS-PAGE, transferred to a PVDF membrane, blocked in 5% Protein Blocking Reagent (Azure) for 1 hr at RT and then probed overnight with primary antibodies at 4 °C. Antibodies included M(LPS/IFNγ) markers iNOS (abcam ab3523; 1:1,000) and MCP1(CST 2029S; 1:500), M(IL-4/IL-10) markers ARG1 (Santa Cruz sc-20150; 1:200), CD206 (abcam ab64693; 1:500), macrophage marker CD68 (abcam ab53444; 1:500) and loading control Gapdh (abcam ab8245; 1:1,000). The following day, blots were washed three times in Protein Wash Buffer (Azure) and probed with HRP-conjugated secondary antibody of the appropriate species at 1:2,500 for one hour at room temperature. Blots were then washed three times in Protein Wash Buffer and once with TBS, developed with Radiance Plus ECL Reagent (Azure), and imaged on the c600 Imager (Azure).

### ELISA and arginase activity assays

Conditioned media for ELISA experiments were generated by culture of 200,000 IC-21 or BMDM in polarization media for two days and then centrifuged at 2,000 g for 10 mins to remove cell debris. MCP1 SimpleStep ELISA (abcam ab208979) was performed on 1:2,000 diluted media in duplicate per manufactures recommendation. Arginase enzymatic activity was quantitated using 1 × 10^6^ polarized macrophages using a colorimetric assay according to manufactures protocol (Sigma-Aldrich).

### Cell proliferation and apoptosis

Metabolically active cells were measured using the Cell Proliferation Reagent WST-1 (Roche) by seeding 5,000 IC-21 or BMDMs in 100 μL per well in triplicate in two identical 96 well plates. After treatment, 10 μL of WST-1 was added to all cell and blank wells of one plate, while the other was changed back into normal macrophage media. Absorbance was measured at 260 nm, following one hour of incubation and a corresponding standard curve was generated with blank-subtracted values normalized to untreated controls. The other plate was processed identically several days later.

### Cytochalasin B

Cytochalasin B (Sigma) was diluted from stock to form 1,000X solution in DMSO and seeded with 100,000 macrophages. Treated and control cells were imaged as above at 24 hours post-seeding, followed by a drug washout with fresh media. At 24- and 48-hour post-removal, cells were again imaged at the same locations.

### Immunofluorescence

Cells were seeded onto glass coverslips for high-resolution imaging. After growth, cells were rinsed in PBS and fixed in warm 4% PFA for 15 min at 37 °C. Cells were blocked in blocking buffer (1% BSA, 5% goat serum, 0.3% triton X-100) for 1 hour at RT, and probed with primary anti-tubulin antibody (abcam ab6160; 1:500) diluted in antibody buffer (1% BSA, 0.3% triton X-100) overnight at 4 °C. Following three washes of PBS, cells were incubated in secondary antibody and phalloidin (1:200) for 1 hour at RT. Cells were again rinsed, mounted on glass overnight in Prolong Gold with DAPI (Invitrogen), and imaged the following day.

### HSPC isolation, transplantation, and cystine measurements

HSPCs were isolated and transplanted into lethally irradiated recipient mice as previously described^16^. In brief, bone marrow cells were flushed from the tibia and femurs of 6 to 8-week old WT, *Ctns*^−/−^ and *Rac2*^−/−^ eGFP mice. HSPCs were isolated by immunomagnetic separation using magnetic beads conjugated to anti-ScaI antibody (Miltenyi Biotec). Isolated HSPCs were directly transplanted via tail vein injection of 100 μL containing 1 × 10^6^ HSPCs into *Ctns*^−/−^ mice subjected to a lethal 7 Gy irradiation the previous day. Engraftment was assessed by percentage of eGFP^+^ cells in peripheral blood at 2- and 4-month post-transplantation. At six months post-transplantation, recipient mice were sacrificed and kidney, liver, and spleen samples were isolated and analyzed by qPCR and mass spectrometry for murine *Ctns* expression and overall cystine load as previously described^15^.

### Statistics

All statistical analyses were performed in Prism (GraphPad). Student’s *t-*test was performed for comparisons of two groups, while one-way analysis of variance (ANOVA) was employed for comparisons of three or more conditions. Outliers were identified and excluded from larger protrusion measurement dataset by the ROUT method^62^. All graphs display mean ± standard deviation.

## Supplementary information

Supplementary Figures

Supplementary Video 1

Supplementary Video 2

Supplementary Video 3

Supplementary Video 4

Supplementary Video 5

## Data Availability

All data sets and ImagePro macros will be shared upon request.
