# Supplementary material for: Macrophage polarization impacts tunneling nanotube formation and intercellular organelle trafficking
Source: Sci Rep. 2019 Oct 10;9:14529. doi: 10.1038/s41598-019-50971-x (PMC6787037; doi:10.1038/s41598-019-50971-x)
Supplement: Supplementary file 1 — Supplementary Figures [file 41598_2019_50971_MOESM1_ESM.docx]

**Macrophage polarization impacts tunneling nanotube formation and intercellular organelle trafficking**

Spencer Goodman, Swati Naphade, Meisha Khan, Jay Sharma, Stephanie Cherqui^*^

Department of Pediatrics, Division of Genetics, University of California, San Diego, La Jolla, California, USA.

**Correspondence to:**

Stephanie Cherqui, Ph.D.

Associate Professor

University of California, San Diego, Department of Pediatrics, Division of Genetics

9500 Gilman Drive, MC 0734, La Jolla, California 92093-0734

Phone: 858-822-1023, Fax: 858-246-1125, email: [scherqui@ucsd.edu](mailto:scherqui@ucsd.edu)

**Supplementary Figures**

**
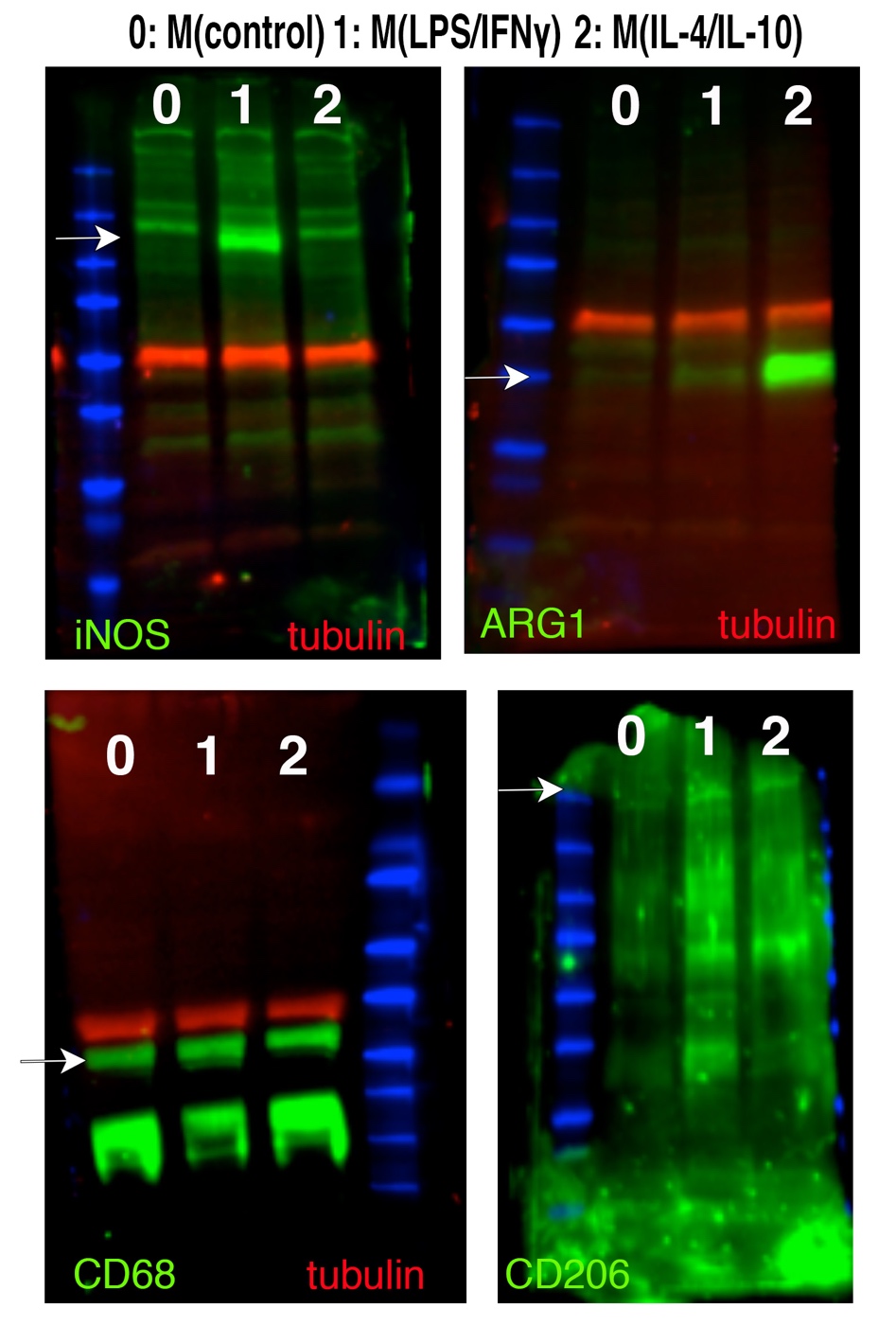
**

**Supplementary Figure S1: Immunoblots of treated BMDMs reveals differences in polarized macrophage protein expression.** Representative entire lanes of western blots against macrophage markers. BMDMs were treated with polarization stimulants for two days prior to harvest of protein in RIPA buffer and quantification by the BCA assay. Equal amounts of protein were then separated at 100V on a 4-20% SDS-PAGE gel before being transferred to PVDF membrane and probed with primary antibodies and chemiluminescent secondary (green). Tubulin was assessed as a loading control using an anti-tubulin-rhodamin antibody (red). Arrows indicate size-expected bands displayed in Figure 1b.

**
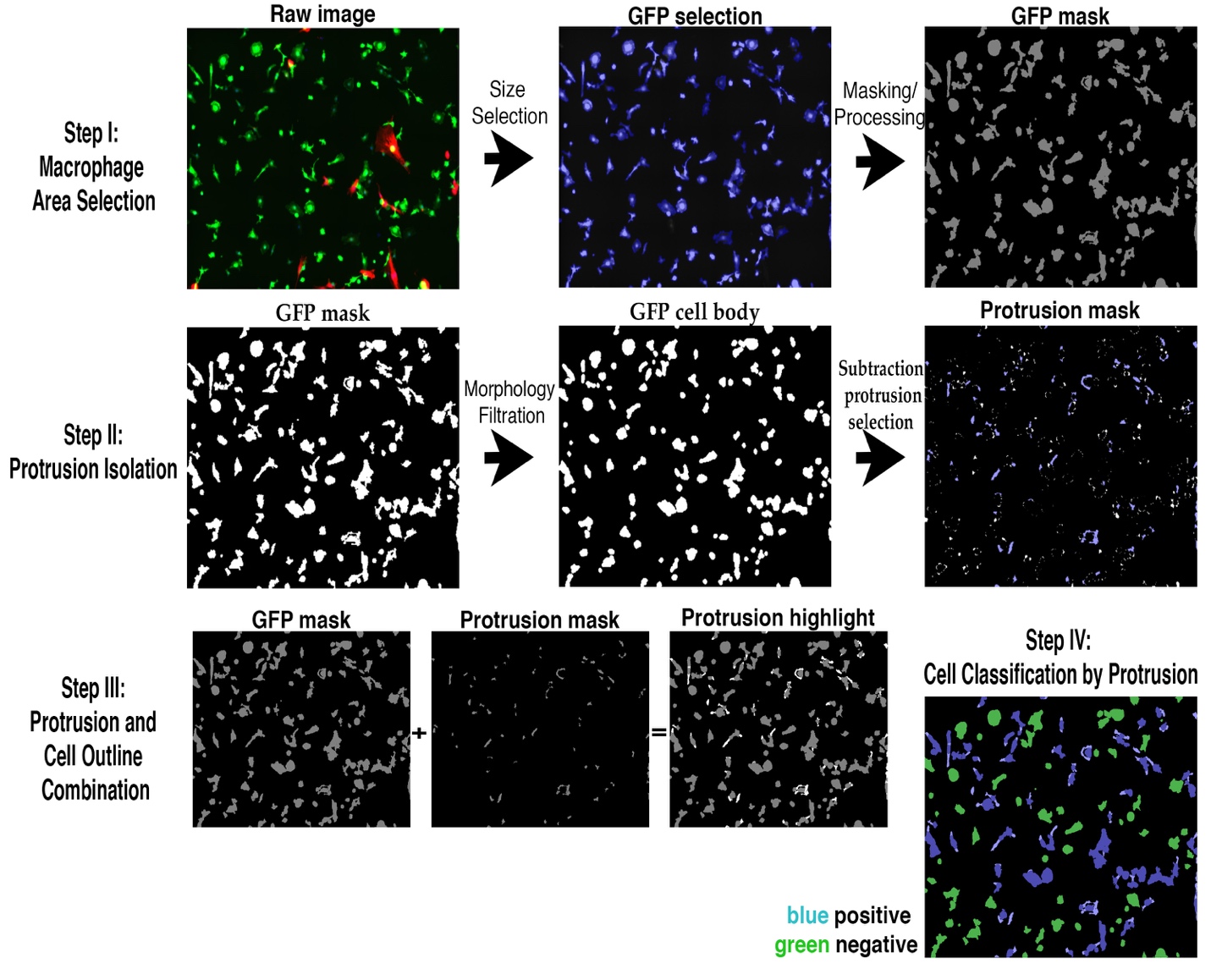
**

**Supplementary Figure S2: Protrusion detection via automated image analysis enables high-throughput quantitation of protrusion frequency.** (**a)** Representative workflow of novel protrusion detection image analysis. Large stitches consisting of an autofocused 5x5 grid of 40x images acquired on the Keyence fluorescent microscope were imported into ImagePro software. Protrusive cells were selected by size (Step I); protrusions were isolated by morphological filtration (Step II); filtered protrusions were overlaid with the original images (Step III); and cells were characterized as protrusion positive (blue) or negative (green) (Step IV). Five stitched regions per experimental condition were analyzed with an automated ImagePro macro system in which imaging and processing settings remained identical for all samples within each experiment.

**Supplementary Videos 1-2**: **3D modeling of eGFP BMDM and IC-21 reveals TNT-like protrusions above surface of dish. (Video 1)** Co-cultures of eGFP^+^ BMDM and *Ctns^-/-^* DsRed^+^ fibroblasts were imaged at 60x to generate a Z-stack and reconstructed into a 3D image in ImagePro. 3D models were generated from the raw signal shown at the start of the video by creating eGFP and DsRed iso-surfaces. **(Video 2)** Co-cultures of eGFP^+^ IC-21 and *Ctns^-/-^* DsRed^+^ fibroblasts were processed in ImagePro as described above.

**
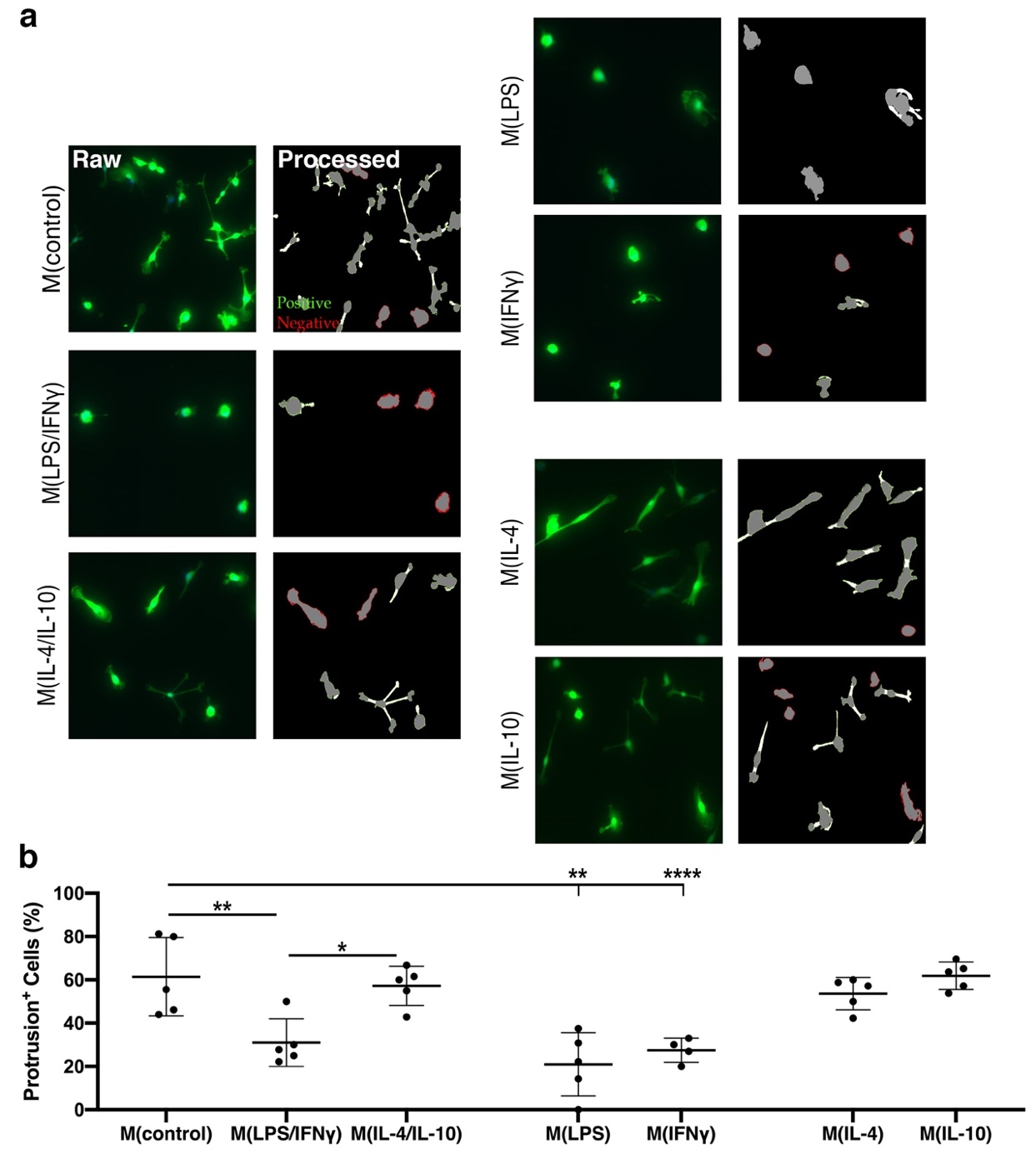
**

**Supplementary Figure S3: BMDM polarization with individual stimulants yields similar change in morphology as combined treatment.** (**a)** Representative raw and processed images of BMDMs treated for two days with either polarization stimulants in combination (left) or each individual stimulant alone (right). (**b**) Quantification of protrusion formation from individually treated BMDMs. Five stitched regions per condition were imaged blind then processed as described in Supplementary Fig. 2. *P* values were determined by one-way ANOVA. * *P* < 0.05, ** *P* < 0.01, *** *P* < 0.001.


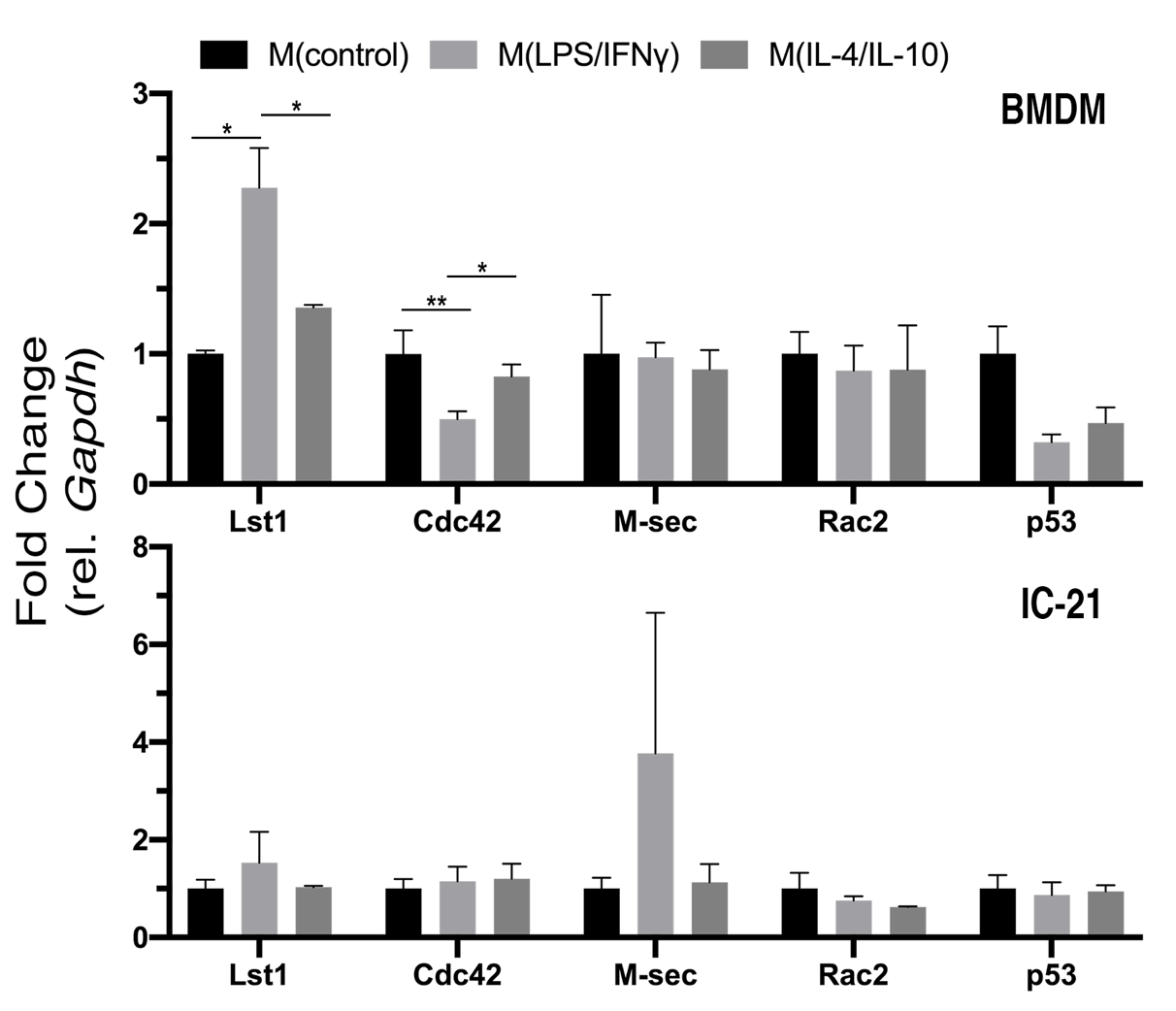


**Supplementary Figure S4: Changes in expression of TNT-related genes following BMDM polarization.** Bar graph showing quantitation of TNT marker mRNA expression relative to the housekeeping control *Gapdh* following 48hrs of M(LPS/IFNγ) or M(IL-4/IL-10) treatment of BMDMs or IC-21 macrophages (n=3). All graphs shown as mean ± SD. *P* values determined by one-way ANOVA * *P* < 0.05, ** *P*< 0.01.


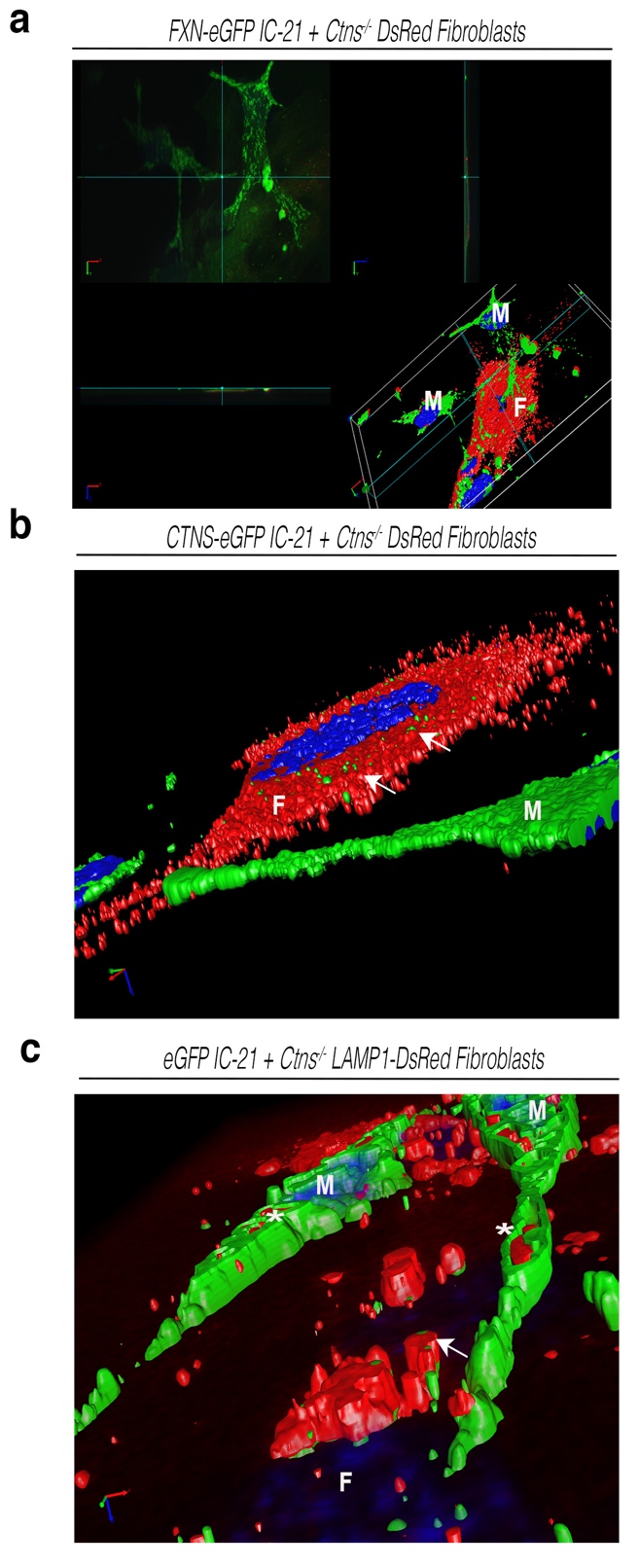


**Supplementary Figure S5: Intercellular transfer of fluorescent molecules confirmed by 3D observations. (a)** Confocal micrograft represented by orthogonal projections in XY (top left), YZ (top right), XZ (bottom left) and 2D representation (bottom right) of co-cultures of frataxin-eGFP IC-21 (labeled M) and *Ctns^-/-^* DsRed^+^ fibroblasts (labeled F). See Supplemental Video 3. (**b**) 2D projection of cystinosin-eGFP IC-21 (M) and *Ctns^-/-^* DsRed fibroblasts (F) depicts eGFP signal within 3D region of DsRed accumulation (arrows). See Supplemental Video 4. (**c**) 2D projection of eGFP IC-21 (M) and *Ctns^-/-^* LAMP2-DsRed fibroblasts (F; LAMP2 being a lysosomal transmembrane protein) depicts DsRed^+^ lysosomes within eGFP protrusions (stars) above the substratum as well eGFP expression within DsRed fibroblast (arrows). See Supplemental Video 5. All images have DAPI shown in blue and were acquired at 60X with 0.3 μM step-size.

**Supplementary Videos 3-5**: **3D-modeling of cystinosin- and frataxin-eGFP IC-21 with DsRed**^+^ **fibroblasts reveal intercellular transfer of lysosomes and mitochondria through macrophage-derived membrane protrusions. (Videos 3-4)** 3D-reconstructions were generated as described previously from co-cultures of either frataxin-eGFP (**Video 3)** or cystinosin-eGFP (**Video 4**) IC-21 macrophages with *Ctns^-/-^* DsRed^+^ fibroblasts. Movie generated from co-cultures of eGFP^+^ IC-21 and *Ctns^-/-^* LAMP2-DsRed fibroblasts (**Video 5**).

**
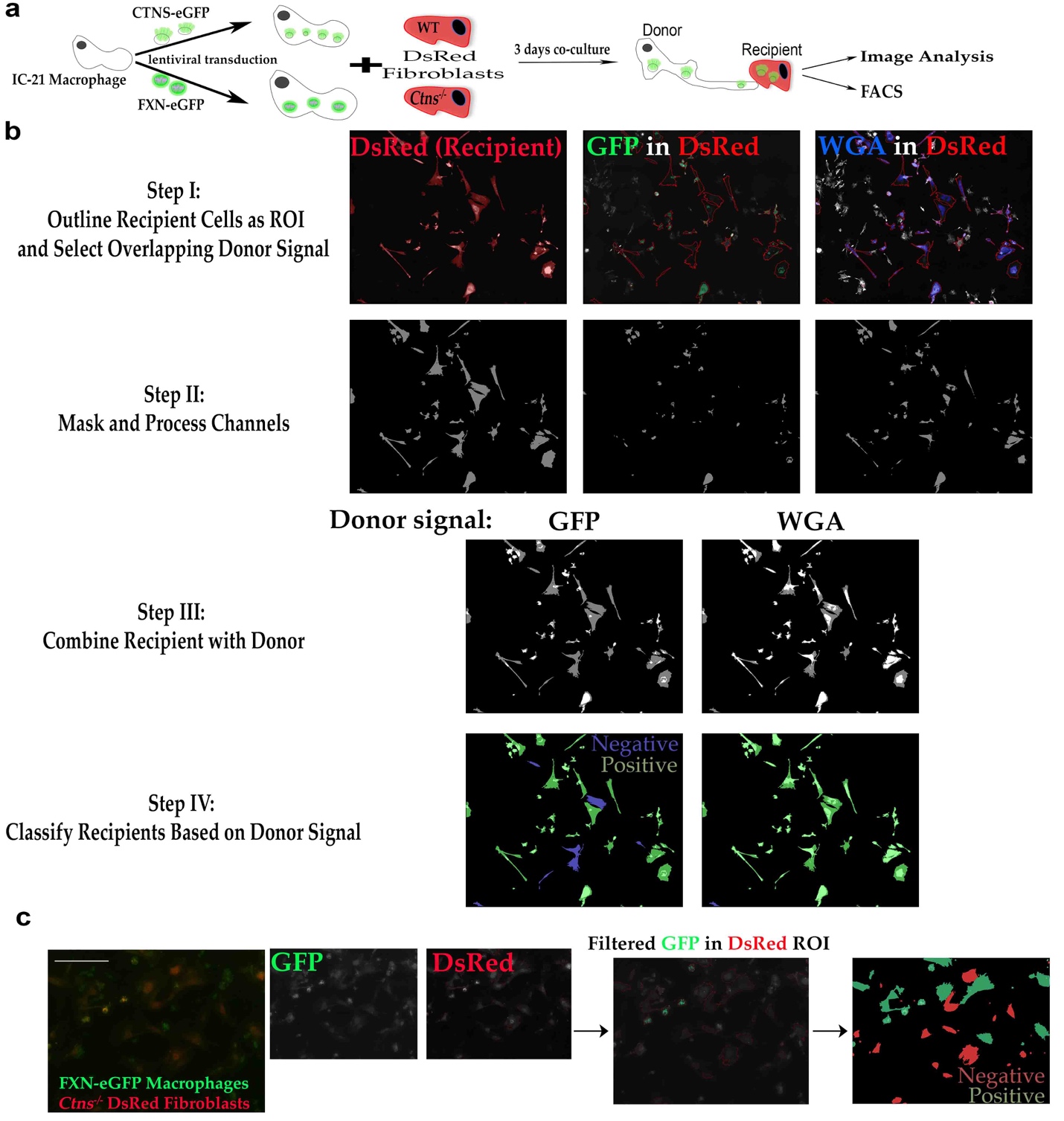
**

**Supplementary Figure S6: Automated detection of fluorescent transfer allows quantitation of intercellular trafficking.** (**a**) Cartoon demonstrating scheme for transfer quantification experiments. (**b**) Representative workflow for transfer quantification. An outline for DsRed^+^ recipient fibroblasts is generated, followed by detection of any donor eGFP or WGA signal (Step 1). Crucially, selected signal was filtered based on size and intensity, ensuring fluorescent background was considered. In addition, any donor signal overlapping cellular border was discounted to reduce overlap artifacts. Filtered donor signal and total recipient ROI were masked and processed (Step 2) and then combined to form an overlay between the two channel masks (Step 3). Finally, cells were classified based on maximum intensity, indicating presence or absence of donor signal (Step 4). (**c**) Representative stitched image taken of frataxin-eGFP macrophages with Ctns^-/-^ DsRed^+^ fibroblasts co-culture. Transfer quantification classifies recipient fibroblasts as either GFP-positive (green) or -negative (red).


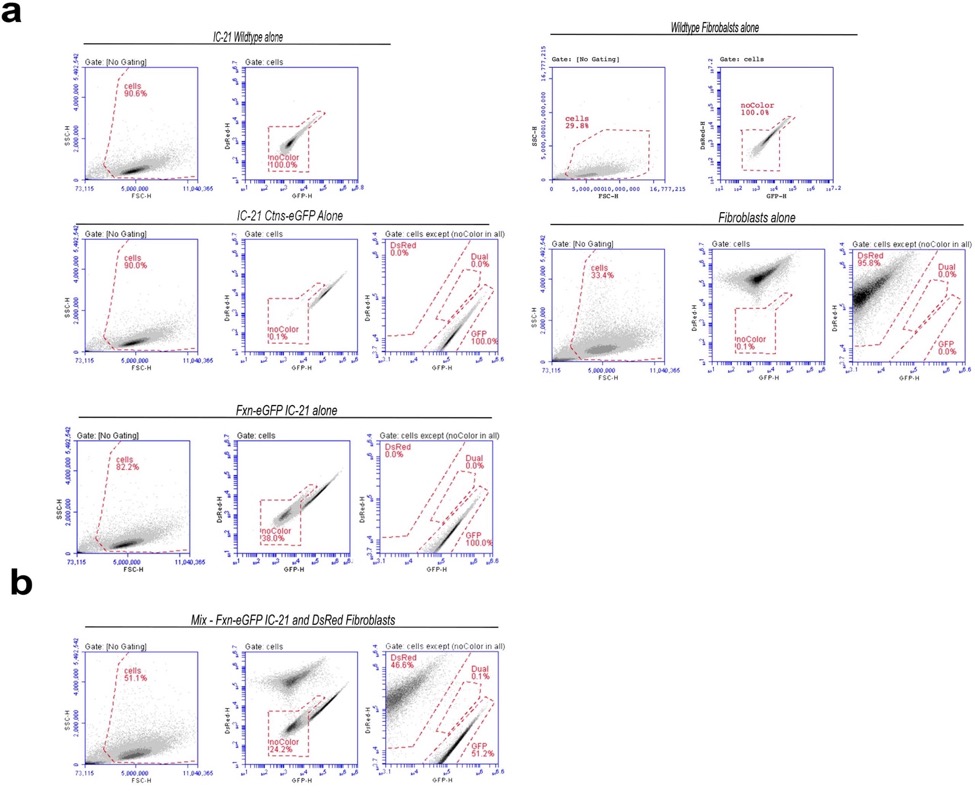


**Supplementary Figure S7: Flow cytometry analysis of cystinosin-eGFP or frataxin-eGFP macrophages co-cultured with DsRed**^+^ **fibroblasts.** (**a**) Flow cytometry dot plots of single-fluorescent cell controls established proper sorting and gating conditions. Sorted samples were first gated on SSC-H and FSC-H to remove debris (left most of each set of FACS plots). Macrophages and fibroblasts without any fluorescence established a “noColor” exclusion gate (middle FACS plot). Finally, eGFP^+^ macrophages (left) and DsRed^+^ fibroblasts alone (right) helped define single-color gates with the intermediate region designated “dual-positive” for eGFP^+^DsRed^+^ cells (right FACS plot). (**b**) Immediately before sorting, populations of eGFP^+^ macrophages and DsRed^+^ fibroblasts cultured separately were mixed together in a single tube. Right plot depicts lack of double-color cells in the “Dual” gate.

*
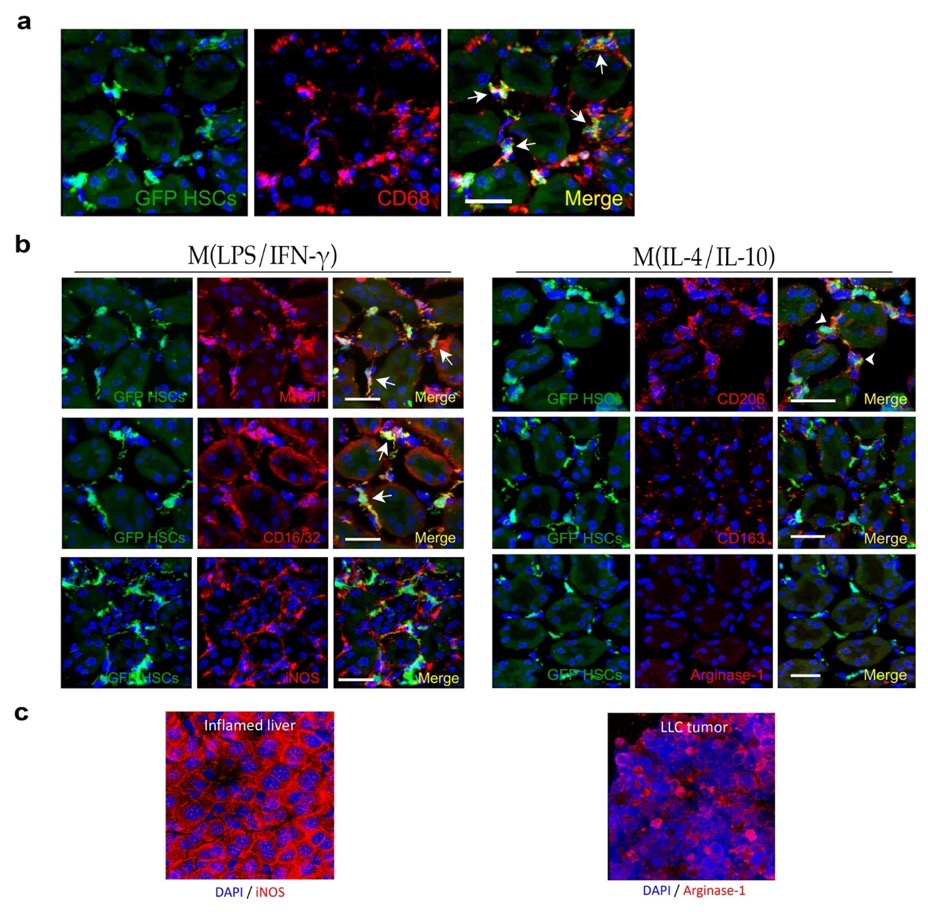
*

**Supplementary Figure S8: HSPC-derived macrophages in kidney appear to preferentially express LPS/IFNγ polarization markers post-transplantation into *Ctns^-/-^* mice.** (**a**) Kidneys isolated from *Ctns^-/-^* mice six months after eGFP^+^ HSPC transplant were fixed, mounted and sectioned in 10 μM slices. Immunofluorescence analysis revealed abundant colocalization (arrows) between the pan-macrophage marker CD68 and eGFP^+^ HSPC progeny. (**b)** Several proinflammatory markers such as MHCII or CD16/32 also co-localized with eGFP^+^ progeny, while colocalization with anti-inflammatory markers was less frequently observed. (**c**) Antibodies were tested for positive activity in other tissues. Scale bars: 10 μM.

**Supplementary Table S1: RT-qPCR Primer Sequences.** List of primers obtained from IDT, resuspended into a 100 μM stock solution and diluted 1:10 before use.
